# Risks of second primary cancers among 584,965 female and male breast cancer survivors in England: a 25-year retrospective cohort study

**DOI:** 10.1016/j.lanepe.2024.100903

**Published:** 2024-04-24

**Authors:** Isaac Allen, Hend Hassan, Walburga Yvonne Joko-Fru, Catherine Huntley, Lucy Loong, Tameera Rahman, Bethany Torr, Andrew Bacon, Craig Knott, Sophie Jose, Sally Vernon, Margreet Lüchtenborg, Joanna Pethick, Katrina Lavelle, Fiona McRonald, Diana Eccles, Eva J.A Morris, Steven Hardy, Clare Turnbull, Marc Tischkowitz, Paul Pharoah, Antonis C. Antoniou

**Affiliations:** a National Disease Registration Service, National Health Service England, London, United Kingdom; b Centre for Cancer Genetic Epidemiology, Department of Public Health and Primary Care, University of Cambridge, Cambridge, United Kingdom; c Division of Genetics and Epidemiology, Institute of Cancer Research, Sutton, United Kingdom; d Health Data Insight CIC, Cambridge, United Kingdom; e Centre for Cancer, Society and Public Health, Comprehensive Cancer Centre, School of Cancer and Pharmaceutical Sciences, King's College London, London, United Kingdom; f Department of Cancer Sciences, Faculty of Medicine, University of Southampton, Southampton, United Kingdom; g Applied Health Research Unit, Big Data Institute, Nuffield Department of Population Health, University of Oxford, Oxford, United Kingdom; h Department of Medical Genetics, Cambridge Biomedical Research Centre, National Institute for Health Research, University of Cambridge, Cambridge, United Kingdom; i Department of Computational Biomedicine, Cedars-Sinai Medical Center, Los Angeles, CA, USA

**Keywords:** Breast cancer, Second primary cancer, Risk, Incidence, Treatment, Pathology, Deprivation, Epidemiology

## Abstract

**Background:**

Second primary cancers (SPCs) after breast cancer (BC) present an increasing public health burden, with little existing research on socio-demographic, tumour, and treatment effects. We addressed this in the largest BC survivor cohort to date, using a novel linkage of National Disease Registration Service datasets.

**Methods:**

The cohort included 581,403 female and 3562 male BC survivors diagnosed between 1995 and 2019. We estimated standardized incidence ratios (SIRs) for combined and site-specific SPCs using incidences for England, overall and by age at BC and socioeconomic status. We estimated incidences and Kaplan–Meier cumulative risks stratified by age at BC, and assessed risk variation by socio-demographic, tumour, and treatment characteristics using Cox regression.

**Findings:**

Both genders were at elevated contralateral breast (SIR: 2.02 (95% CI: 1.99–2.06) females; 55.4 (35.5–82.4) males) and non-breast (1.10 (1.09–1.11) females, 1.10 (1.00–1.20) males) SPC risks. Non-breast SPC risks were higher for females younger at BC diagnosis (SIR: 1.34 (1.31–1.38) <50 y, 1.07 (1.06–1.09) ≥50 y) and more socioeconomically deprived (SIR: 1.00 (0.98–1.02) least deprived quintile, 1.34 (1.30–1.37) most).

**Interpretation:**

Enhanced SPC surveillance may benefit BC survivors, although specific recommendations require more detailed multifactorial risk and cost-benefit analyses. The associations between deprivation and SPC risks could provide clinical management insights.

**Funding:**

CRUK Catalyst Award CanGene-CanVar (C61296/A27223). Cancer Research UK grant: PPRPGM-Nov 20∖100,002. This work was supported by core funding from the 10.13039/501100018956NIHR Cambridge Biomedical Research Centre (NIHR203312)]. The views expressed are those of the author(s) and not necessarily those of the NIHR or the Department of Health and Social Care.

**Research in context**

**Evidence before this study**

There have been multiple studies assessing second primary cancer risks following breast cancer in females, and several such studies in males. We conducted two systematic reviews and meta-analyses of second primary cancer (SPC) risks following breast cancer (BC) in females and males. In each of these reviews, we searched PubMed, Embase, and Web of Science for studies reporting standardized incidence ratios (SIRs) for non-breast SPC development published by March 2022. We also reviewed the bibliographies of the studies found in these searches. We found that although non-breast SPC risks were elevated for BC survivors of either gender, few studies were able to examine the effects of patient demographics, BC pathology, or treatments for the first BC on SPC risks, and studies that did were often small. SPC risk estimates were inconsistent between studies and many analyses for male BC survivors were underpowered. Finally, studies rarely accounted for surgeries at potential SPC sites in their censoring processes, despite prophylactic or curative partial or full resections of the breast, and prophylactic partial or full resections of the ovary or endometrium, both being commonly performed in breast cancer survivors and having clear implications for SPC risks at these sites.

**Added value of this study**

Here, for the first time, we used data on 581,403 female and 3562 male BC survivors from the National Health Service England, with linked electronic health records and comprehensive data quality control, to assess SPC risks and the variation in SPC risks by demographic factors, BC pathology and BC treatment. This is the largest study to date to examine SPC risks in BC survivors of either gender. The results show that both female and male BC survivors have significantly elevated SPC risks at all sites combined, all non-breast sites combined, and specific sites including the contralateral breast, ovary, endometrium, and prostate. To our knowledge, our study is the first to demonstrate that socioeconomically deprived BC survivors are at greater risk of SPCs compared to less deprived BC survivors. The study has provided increased precision in SPC risk estimates in both women and men, further elucidating the factors contributing to the variability in SPC risks.

**Implications of all the available evidence**

BC survivors may benefit from enhanced surveillance for SPCs, particularly at the contralateral breast, endometrium, and prostate, although specific recommendations would require separate cost-benefit analyses. This study may also aid risk stratification for SPCs in BC survivors, since we found significant evidence for variation in SPC risks in females by the age and calendar year at first BC diagnosis, the size, grade, morphology, estrogen receptor status or Human Epidermal growth factor Receptor 2 status of the first BC, the administration of chemotherapy, radiotherapy or hormonal therapy for the first BC, socioeconomic deprivation, and ethnicity. Finally, this study demonstrates the opportunities arising from the use of population-scale, comprehensive, linked electronic health records datasets, while also enabling us to account for the possible sources of variability in risk. Future studies should aim to examine the influence of deprivation-associated factors such as smoking or obesity on the risks of second primaries following BC.

## Introduction

Breast cancer (BC) was the most common cancer diagnosed globally in 2020.^1^ 5-year survival has increased from 81% to 87% for those diagnosed between 2001 and 2005 and 2013 and 2017 in England.^2^ Male BC accounts for <1% of United Kingdom (UK) BC diagnoses,^3^ but follows similar trends.^4^ Accurate estimates of second primary cancer (SPC) risks following BC, and the variation in these risks by sociodemographic factors, first BC pathology, and treatments administered for the first BC, are thus necessary to inform the clinical management of a growing number of BC survivors.

Recent systematic reviews estimated female and male non-breast SPC risks following BC as 24% and 27% greater than population-level,^5^^,^^6^ with greater relative increases in those diagnosed with BC below age 50.^5^^,^^6^ Female thyroid, endometrial, ovary, kidney, oesophagus, melanoma, leukaemia, lung, stomach, and bladder SPCs risks were elevated,^5^ whereas males were at increased thyroid, pancreatic, and colorectal SPC risks.^6^ These studies produced disparate risk estimates and relied predominantly on population-based cancer registries without linkages to other data, so were unable to reliably assess risk variation by sociodemographic factors, BC pathology, and BC treatment.

We assessed SPC risks in 581,403 female and 3562 male BC survivors diagnosed between 1995 and 2019 using the National Cancer Registration Dataset (NCRD),^7^ a population-scale dataset with 98–99% complete case ascertainment.^8^ This is the largest study performed to date in BC survivors of either gender and the first to examine the risks in the NCRD. Participant data were linked to obtain comprehensive data on socioeconomic status, BC pathology, BC treatment, curative and prophylactic surgeries, and predominantly self-reported ethnicity. We aimed to estimate combined and site-specific relative and absolute SPC risks in both genders, and assess variability in risks by socioeconomic factors, tumour characteristics and treatments, at sites at particularly elevated risk following BC: the contralateral breast,^9^ endometrium,^5^ ovary,^5^ and all non-breast sites combined.^5^^,^^6^

## Methods

### Study population

Data originated from the National Cancer Registration Dataset (NCRD),^7^ Hospital Episode Statistics Admitted Patient Care (HES APC)^10^ and HES Outpatients (HES OP) datasets within the National Health Service (NHS) England (NHSE). We constructed a retrospective cohort of those diagnosed with invasive first primary BC between 1st January 1995 and 31st Dec 2019. To ensure that the first BC was invasive and non-metastatic, BC survivors with missing staging data were filtered from the cohort. Diagnosis date, person-stated gender (self-declared or inferred by observation, henceforth ‘gender’), age, socioeconomic deprivation, BC pathology, BC laterality, chemotherapy, radiotherapy, hormonal therapy, SPC diagnoses, whether BC survivors had permanently left the UK (henceforth ‘embarked’), and death data were drawn from the NCRD. Surgical procedures data were extracted from the HES APC and HES OP datasets. The data were linked using unique patient and tumour identifiers. Descriptions of the datasets and quality control processes are available in the Supplementary Material.

### Statistical analyses

The outcome of interest was an invasive SPC diagnosis. Follow-up lasted from 365 days after BC diagnosis to the earliest of death, embarkation, invasive SPC diagnosis, or the 31st of December 2020. Surgeries at the contralateral breast, endometrium, and ovary were also considered censoring events when evaluating site-specific risks (Supplementary Material).

We defined SPC sites using the ICD-10 code groups employed by Cancer Research UK.^11^ We did not consider contralateral BC (CBC)s observed within 92 days of BC diagnosis or any ipsilateral BCs as SPCs, to avoid misclassifications of first BC recurrences. We did not consider non-melanoma skin cancer a SPC. Follow-up would thus continue after recorded diagnoses of ipsilateral second BCs, CBCs within 92 days of first BC diagnosis, or non-melanoma skin cancers. We did not consider cancers diagnosed from death certificates only as SPCs.

### Standardised incidence ratios, incidences and cumulative risks

We estimated standardized incidence ratios (SIRs), incidence rates (IRs) and cumulative risks (CRs) for SPCs. We estimated SIRs for SPCs at all sites combined, all non-breast sites combined, and the 20 most common cancer sites diagnosed between 2016 and 2018 in the UK excluding cancer of unknown primary,^11^ by comparing observed SPC counts in our cohort to expected cancer counts. We examined the risks of myeloid and non-myeloid leukaemia SPCs separately. We calculated expected counts based on age-, calendar year-, cancer site, and gender-specific cancer incidences for England, excluding cancers diagnosed from death certificates only.^12^ We estimated unstratified SIRs for SPCs in females and males, as well as SIRs for SPCs in females stratified by age at BC diagnosis (<50 y and ≥50 y) and by indices of multiple deprivation (IMD) quintile, a measure of socioeconomic deprivation based on region of residence at first BC diagnosis.^13^ We stratified the male SIRs for all non-breast SPCs by age at BC diagnosis and IMD quintile, but did not stratify SIRs for specific sites in males due to low SPC counts. Finally, we stratified SIRs for myeloid leukaemia SPCs in females by receipt of chemotherapy, and stratified SIRs for endometrial SPCs by calendar year of first BC diagnosis and receipt of hormonal therapy.

We estimated incidences per 10,000 person-years (py) for five-year follow-up periods (0–5 y, 5–10 y, 10–15 y, 15–20 y, 20–25 y) for all non-breast SPCs combined. We also estimated incidences for contralateral breast, ovarian and endometrial SPCs and estimated 25-year CRs using Kaplan–Meier analyses. All incidences and CRs were stratified by age at BC diagnosis (<50 y and ≥50 y).

### Associations with socio-demographic, tumour, and treatment characteristics

We used Cox proportional hazards (CPH) modelling to estimate hazard ratios (HRs) for associations between non-breast, contralateral breast (females only), endometrial and ovarian SPC risks and patient characteristics (age at BC diagnosis, calendar year at BC diagnosis, ethnicity, and IMD quintile), tumour characteristics (size, number of nodes involved, grade, morphology, ER status, and HER2 status) and treatment (chemotherapy, radiotherapy, and hormonal therapy administered no later than 1 y post-BC diagnosis). Records of borderline ER or HER2 status were regarded as missing information due to their rarity and were imputed. We imputed missing data for ethnicity and for the size, number of nodes involved in, grade, morphology, ER status, and HER2 status of the first breast tumour with multiple imputation by chained equations^14^ separately for females and males (Supplementary Material), with degrees of missingness for these variables ranging from <0.1% for first BC morphology to 55.3% for first BC ER status in females and from 0% for first BC morphology to 54.8% for first BC HER2 status in males (Supplementary Material).

We estimated HRs for a given target variable by first fitting CPH models adjusted only for age at BC diagnosis. We then assessed the correlations between the target variable and all other variables found significant in this first set of models using the chi-squared test to compare two nominal variables, Spearman's rank test to compare two ordinal variables, and the Kruskal–Wallis test to compare an ordinal to a nominal variable. Finally, we fitted a second, multivariable CPH model adjusted for age and all other variables found to be both significantly correlated with the target variable and significantly associated with SPC risks when adjusted only for age in the first set of models. The only exception to this process was when estimating HRs for the receipt of chemotherapy, radiotherapy, or hormonal therapy. If any of the three treatments were found to be significant when adjusted only for age, all three were included in the multivariable model. This was done to account for the administration of different types of BC treatment as part of the same cycle. For each variable, we visually inspected a plot of the logarithm of the negative logarithm of the survival function across follow-up time against relevant reference categories to assess any deviation from the proportional hazards assumption.

All analyses were performed separately by gender. All statistical analyses were performed in R, with details of the version and packages used in the Supplementary Material.

### Role of the funding source

The study sponsors had no role in study design, in data collection, analysis, and interpretation, in the writing of this article, or in the decision to submit this article for publication.

## Results

### Cohort description

**Fig. 1:**
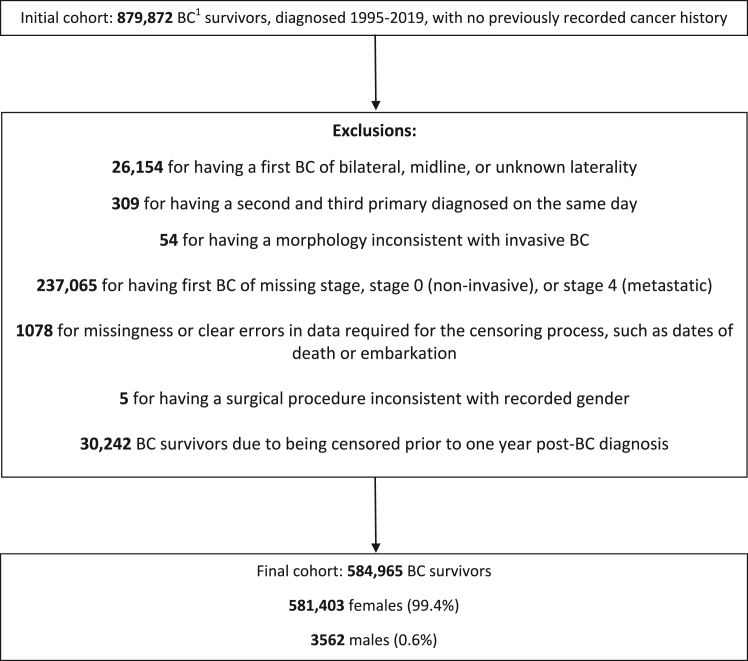
**Cohort assembly**. 1: Breast Cancer. **Note:** When examining the site-specific risks of second primary CBC, a further 8721 BC survivors were filtered from the cohort (15 male, 8706 female) due to having received a censoring surgery before the start of follow-up. When examining the site-specific risks of endometrial SPCs, a further 17,081 BC survivors were filtered from the cohort (all female) due to having received a censoring surgery before the start of follow-up. When examining the site-specific risks of ovarian SPCs, a further 9425 BC survivors were filtered from the cohort (all female) due to having received a censoring surgery before the start of follow-up.

Following exclusions (Fig. 1), the cohort consisted of 581,403 females and 3562 males. Participants were predominantly aged 50 or over at first BC diagnosis (females: 78%, males: 92%), diagnosed with BC between 2010 and 2019 (females: 55%, males: 56%) and of White ethnicity (females: 86%, males: 84%). There were 52,620 and 504 SPCs diagnosed among females and males respectively. The mean ages at first primary BC diagnosis were 61 years for females and 67 years for males and the corresponding median lengths of follow-up were 5.5 and 4.4 years. Further details are in Table 1.

**Table 1:** Cohort description.

	Female cohort (all)		Female cohort (with a SPC^a^)		Male cohort (all)		Male cohort (with a SPC)	
	Mean/median/IQRs^b^: Age at BC^c^ diagnosis (years): 61, 60, 20 Follow-up time (years): 7.4, 5.5, 8.4		Mean/median/IQRs: Age at BC diagnosis (years): 61, 61, 18 Follow-up time (years): 7.5, 6.3, 8.6		Mean/median/IQRs: Age at BC diagnosis (years): 67, 68, 16 Follow-up time (years): 6.0, 4.4, 6.4		Mean/median/IQRs: Age at BC diagnosis (years): 69, 69, 12 Follow-up time (years): 5.7, 4.0, 7.3	
	Number BC (%^d^)	Total FU^e^ (py^f^) (%^g^)	Number BC (%)	Total FU (py) (%)	Number BC (%)	Total FU (py) (%)	Number BC (%)	Total FU (py) (%)
**Age at first BC diagnosis**								
Under 50	127,048 (21.9)	1,094,793 (25.5)	8772 (16.7)	79,011 (20.0)	293 (8.2)	2335 (11.0)	14 (2.8)	110 (3.8)
50 or over	454,355 (78.1)	3,191,071 (74.5)	43,848 (83.3)	317,009 (80.0)	3269 (91.8)	18,932 (89.0)	490 (97.2)	2765 (96.2)
**Year of first BC diagnosis**								
1995–1999	98,972 (17.0)	1,243,982 (29.0)	16,341 (31.1)	175,508 (44.3)	635 (17.8)	5880 (27.6)	147 (29.2)	1259 (43.8)
2000–2004	93,877 (16.1)	1,095,236 (25.6)	14,319 (27.2)	125,922 (31.8)	534 (15.0)	5008 (23.5)	111 (22.0)	793 (27.6)
2005–2009	70,068 (12.1)	684,389 (16.0)	8564 (16.3)	54,073 (13.7)	387 (10.9)	3097 (14.6)	68 (13.5)	396 (12.8)
2010–2014	136,865 (23.5)	842,092 (19.6)	9187 (17.5)	33,570 (8.5)	881 (24.7)	4882 (23.0)	123 (24.4)	379 (13.2)
2015–2019	181,621 (31.2)	420,164 (9.8)	4209 (8.0)	6946 (1.8)	1125 (31.6)	2401 (11.3)	55 (10.9)	75 (2.6)
**Ethnicity**								
White	497,621 (85.6)	3,698,702 (86.3)	49,196 (93.5)	374,861 (94.7)	2999 (84.2)	18,184 (85.5)	468 (92.9)	2728 (94.9)
Asian	14,025 (2.4)	85,475 (2.0)	790 (1.5)	5798 (1.5)	112 (3.1)	646 (3.0)	7 (1.4)	39 (1.3)
Black	8240 (1.4)	46,677 (1.1)	483 (0.9)	3386 (0.9)	59 (1.7)	251 (1.2)	9 (1.8)	34 (1.2)
Chinese	1518 (0.3)	9984 (0.2)	66 (0.1)	465 (0.1)	4 (0.1)	21 (<0.1)	1 (0.2)	8 (0.2)
Mixed	2347 (0.4)	14,013 (0.3)	147 (0.3)	1168 (0.3)	15 (0.4)	85 (0.4)	2 (0.4)	7 (0.3)
Other	6093 (1.0)	34,719 (0.8)	328 (0.6)	2350 (0.6)	33 (0.9)	175 (0.8)	3 (0.6)	15 (0.5)
Data missing	51,559 (8.9)	396,293 (9.2)	1610 (3.1)	7991 (2.0)	340 (9.5)	1905 (9.0)	14 (2.8)	45 (1.6)
**Indices of Multiple Deprivation quintile**^h^								
1 (most deprived)	86,043 (14.8)	586,188 (13.7)	8178 (15.5)	58,798 (14.8)	537 (15.1)	3096 (14.6)	66 (13.1)	394 (13.7)
2	103,326 (17.8)	737,078 (17.2)	9285 (17.6)	68,551 (17.3)	641 (18.0)	3638 (17.1)	94 (18.7)	462 (16.1)
3	121,561 (20.9)	891,441 (20.8)	10,908 (20.7)	81,742 (20.6)	758 (21.3)	4508 (21.2)	108 (21.4)	545 (19.0)
4	132,562 (22.8)	1,000,633 (23.3)	11,951 (22.7)	90,430 (22.8)	763 (21.4)	4715 (22.2)	112 (22.2)	702 (24.4)
5 (least deprived)	137,911 (23.7)	1,070,593 (25.0)	12,298 (23.4)	96,499 (24.4)	863 (24.2)	5310 (25.0)	124 (24.6)	773 (26.9)
**Has had chemotherapy**^i^								
Negative^j^	404,945 (69.6)	3,064,246 (71.5)	39,958 (75.9)	301,256 (76.1)	2761 (77.5)	16,772 (78.9)	409 (81.2)	2317 (80.6)
Positive	176,458 (30.4)	1,221,617 (28.5)	12,662 (24.1)	94,763 (23.9)	801 (22.5)	4496 (21.1)	95 (18.8)	558 (19.4)
**Has had radiotherapy**^i^								
Negative^k^	240,691 (41.4)	1,799,483 (42.0)	22,619 (43.0)	171,285 (43.3)	1996 (56.0)	11,577 (54.4)	280 (55.6)	1578 (54.9)
Positive	340,712 (58.6)	2,486,381 (58.0)	30,001 (57.0)	224,734 (56.7)	1566 (44.0)	9691 (45.6)	224 (44.4)	1298 (45.1)
**Has had hormonal therapy**^i^								
Negative^l^	317,875 (54.7)	2,289,614 (53.4)	27,279 (51.8)	203,226 (51.3)	1732 (48.6)	10,250 (48.2)	242 (48.0)	1405 (48.9)
Positive	263,528 (45.3)	1,996,249 (46.6)	25,341 (48.2)	192,793 (48.7)	1830 (51.4)	11,018 (51.8)	262 (52.0)	1470 (51.1)
**Has had contralateral breast surgery**^m^								
No^n^	529,165 (91.0)	3,897,761 (90.9)	48,172 (91.5)	362,834 (91.6)	3385 (95.0)	20,169 (94.8)	480 (95.2)	2757 (95.9)
Yes (before first BC diagnosis)	7259 (1.2)	45,474 (1.1)	546 (1.0)	3547 (0.9)	6 (0.2)	52 (0.2)	2 (4.6)	2 (<0.1)
Yes (after/at first BC diagnosis)	44,979 (7.7)	342,629 (8.0)	3902 (7.4)	29,639 (7.5)	171 (4.8)	1047 (4.9)	22 (4.4)	116 (4.0)
**Has had endometrial surgery**^m^								
No^o^	549,598 (94.5)	4,013,722 (93.7)	49,210 (93.5)	368,414 (93.0)	–	–	–	–
Yes (before first BC diagnosis)	16,921 (2.9)	96,795 (2.3)	963 (1.8)	5254 (1.3)	–	–	–	–
Yes (after/at first BC diagnosis)	14,884 (2.6)	175,347 (4.1)	2447 (4.7)	22,352 (5.6)	–	–	–	–
**Has had bilateral ovarian surgery**^m^								
No^p^	552,529 (95.0)	4,038,718 (94.2)	49,394 (93.9)	370,191 (93.5)	–	–	–	–
Yes (before first BC diagnosis)	9167 (1.6)	51,472 (1.2)	524 (1.0)	2917 (0.7)	–	–	–	–
Yes (after/at first BC diagnosis)	19,707 (3.4)	195,673 (4.6)	2702 (5.1)	22,912 (5.8)	–	–	–	–
**Totals**								
Total BC cases/FU (py)	581,403 (100)	4,285,864 (100)	52,620 (100)	396,020 (100)	3562 (100)	21,268 (100)	504 (100)	2876 (100)

a Second Primary Cancer.

b Inter-Quartile Range.

c Breast Cancer.

d Percentage of BC cases.

e Follow-up.

f Person-Years.

g Percentage of follow-up years.

h To avoid confusion, it should be noted that Indices of Multiple Deprivation quintiles are recorded for the entire population of the United Kingdom based on geographic area, explaining why there are not exact fifths of the cohort recorded in each Indices of Multiple Deprivation quintile.

i By start of follow-up.

j No record of chemotherapy found in the National Cancer Registration Dataset.

k No record of radiotherapy found in the National Cancer Registration Dataset.

l No record of hormonal therapy found in the National Cancer Registration Dataset.

m By end of follow-up.

n No record of contralateral breast surgery found in the Hospital Episode Statistics Admitted Patient Care dataset.

o No record of endometrial surgery found in the Hospital Episode Statistics Admitted Patient Care dataset.

p No record of bilateral ovarian surgery found in the Hospital Episode Statistics Admitted Patient Care dataset.

### Standardized incidence ratios

Female BC survivors were at elevated SPC risks at all sites combined (SIR: 1.25 (95% CI: 1.24–1.26)), the contralateral breast (SIR: 2.02 (95% CI: 1.99–2.06)), and all non-breast sites combined (SIR: 1.10 (95% CI: 1.09–1.11)). Site-specific SIR point estimates ranged between 0.87 and 2.02 and were elevated for all SPCs except for non-Hodgkin's lymphoma, myeloma, and brain and central nervous system (Table 2). The largest SIRs for non-breast sites were observed for endometrial (SIR: 1.87 (95% CI: 1.82–1.93)), myeloid leukaemia (SIR: 1.58 (95% CI: 1.47–1.70)), and ovarian (SIR: 1.25 (95% CI: 1.21–1.30)) SPCs.

**Table 2:** Standardized incidence ratios for second primaries, unstratified and stratified by age at first breast cancer diagnosis (female survivors only).

Cancer site	Female–unstratified		Female: age <50 at first BC^a^ dx^b^		Female: age≥50 at first BC dx		Male–unstratified	
	SIR^c^ (95% CI^d^)	O^e^	SIR	O	SIR	O	SIR	O
All sites combined	1.25 (1.24–1.26)	51,767	1.86 (1.82–1.90)	8466	1.17 (1.16–1.18)	43,301	1.15 (1.05–1.26)	503
All non-breast sites combined	1.10 (1.09–1.11)	38,419	1.34 (1.31–1.38)	4548	1.07 (1.06–1.09)	33,871	1.10 (1.00–1.20)^f^	479
Contralateral breast	2.02 (1.99–2.06)	13,348	3.36 (3.26–3.47)	3918	1.73 (1.70–1.77)	9430	55.4 (35.5–82.4)	24
Lung	1.06 (1.03–1.08)	7083	1.52 (1.41–1.63)	729	1.02 (1.00–1.05)	6354	0.74 (0.55–0.98)	49
Colorectum	1.08 (1.06–1.11)	6322	1.18 (1.08–1.29)	520	1.07 (1.05–1.10)	5802	1.17 (0.91–1.48)	69
Endometrium	1.87 (1.82–1.93)	4842	1.92 (1.77–2.07)	630	1.87 (1.81–1.93)	4212	–	–
Ovary	1.25 (1.21–1.30)	2513	1.73 (1.58–1.89)	468	1.18 (1.13–1.23)	2045	–	–
Melanoma	1.12 (1.07–1.17)	1976	1.15 (1.03–1.28)	357	1.11 (1.06–1.17)	1619	1.14 (0.65–1.85)	16
Pancreas	1.18 (1.12–1.23)	1817	1.65 (1.40–1.92)	162	1.15 (1.09–1.20)	1655	1.62 (0.99–2.50)	20
Non-Hodgkin's lymphoma	0.93 (0.89–0.98)	1712	1.05 (0.90–1.21)	191	0.92 (0.88–0.97)	1521	1.12 (0.67–1.78)	18
Kidney	1.11 (1.05–1.17)	1424	1.30 (1.12–1.52)	170	1.08 (1.02–1.15)	1254	1.27 (0.77–1.95)	20
Blood (non-myeloid leukaemia)	1.07 (0.99–1.15)	693	1.31 (1.01–1.68)	64	1.05 (0.97–1.14)	629	0.86 (0.34–1.77)	7
Blood (myeloid leukaemia)	1.58 (1.47–1.70)	705	2.26 (1.85–2.73)	105	1.50 (1.38–1.63)	600	1.31 (0.48–2.84)	6
Head and neck	1.09 (1.03–1.16)	1059	1.26 (1.08–1.46)	180	1.06 (0.99–1.14)	879	0.60 (0.26–1.17)	8
Bladder	1.03 (0.97–1.09)	1002	1.21 (0.92–1.57)	57	1.02 (0.96–1.09)	945	0.34 (0.15–0.67)	8
Oesophagus	1.06 (0.99–1.13)	986	1.35 (1.07–1.69)	78	1.04 (0.97–1.11)	908	0.75 (0.38–1.35)	11
Stomach	1.19 (1.11–1.27)	947	1.41 (1.09–1.78)	69	1.17 (1.10–1.25)	878	1.12 (0.63–1.85)	15
Blood (myeloma)	0.92 (0.85–0.99)	708	0.90 (0.68–1.17)	55	0.92 (0.85–1.00)^f^	653	0.76 (0.28–1.65)	6
Liver	1.05 (0.97–1.13)	599	1.04 (0.75–1.41)	42	1.05 (0.96–1.14)	557	1.02 (0.44–2.00)	8
Brain and central nervous system	0.87 (0.80–0.96)	489	1.00 (0.79–1.25)	78	0.85 (0.77–0.94)	411	0.81 (0.22–2.06)	4
Thyroid	1.20 (1.09–1.32)	460	1.29 (1.08–1.52)	137	1.17 (1.05–1.30)	323	3.74 (1.01–9.58)	4
Prostate	–	–	–	–	–	–	1.58 (1.36–1.82)	190

a Breast Cancer.

b Diagnosis.

c Standardized Incidence Ratio.

d Confidence Interval.

e Observed count of second primaries.

f Although the lower/upper confidence interval boundary was rounded to 1.00, the result was significant (p < 0.05).

SIRs for SPCs at all sites combined were higher for females diagnosed with BC when younger (<50 y: SIR: 1.86 (95% CI: 1.82–1.90), ≥50 y: SIR: 1.17 (95% CI: 1.16–1.18)) as well as at all non-breast sites combined (<50 y: SIR: 1.34 (95% CI: 1.31–1.38), ≥50 y: SIR: 1.07 (95% CI: 1.06–1.09)). There were clear differences in SIRs by age at BC diagnosis for contralateral breast (<50 y: SIR: 3.36 (95% CI: 3.26–3.47), ≥ 50 y: SIR: 1.73, (95% CI: 1.70–1.77)), lung (<50 y: SIR: 1.52 (95% CI: 1.41–1.63), ≥ 50 y: SIR: 1.02 (95% CI: 1.00–1.05)), pancreatic (<50 y: SIR: 1.65 (95% CI: 1.40–1.92), ≥ 50 y: SIR: 1.15 (95% CI: 1.09–1.20)), ovarian (<50 y: SIR: 1.73 (95% CI: 1.58–1.89), ≥ 50 y: SIR: 1.18 (95% CI: 1.13–1.23)), and myeloid leukaemia (<50 y: SIR: 2.26 (95% CI: 1.85–2.73), ≥ 50 y: SIR: 1.50 (95% CI: 1.38–1.63)) SPCs.

SPC risks were elevated for females in more deprived IMD quintiles (Table 3), with SIRs for SPCs at all sites combined of 1.16 (95% CI: 1.14–1.18) and 1.46 (95% CI: 1.43–1.49) for the least deprived quintile (LDQ, quintile 5) and most deprived quintile (MDQ, quintile 1). These differences were primarily driven by non-breast sites, where the corresponding SIRs were 1.00 (95% CI: 0.98–1.02) and 1.34 (95% CI: 1.30–1.37). There were no marked differences in SIRs for CBC by IMD quintile. The clearest differences were for lung (LDQ: SIR: 0.70 (95% CI: 0.66–0.74), MDQ: SIR: 1.86 (95% CI: 1.77–1.95)), kidney (LDQ: SIR: 0.96 (95% CI: 0.86–1.08), MDQ: SIR: 1.37 (95% CI: 1.20–1.56)), head and neck (LDQ: SIR: 0.93 (95% CI: 0.81–1.06), MDQ: SIR: 1.38 (95% CI: 1.19–1.60)), bladder (LDQ: SIR: 0.85 (95% CI: 0.73–0.97), MDQ: SIR: 1.30, (95% CI: 1.11–1.51)), oesophagus (LDQ: SIR: 0.93 (95% CI: 0.81–1.06), MDQ: SIR: 1.44 (95% CI: 1.24–1.67)) and stomach (SIR: 0.95 (95% CI: 0.82–1.10), MDQ: SIR: 1.69 (95% CI: 1.45–1.96)) SPCs. We also found increased second primary melanoma risks in less deprived BC survivors (LDQ: SIR: 1.41 (95% CI: 1.30–1.52), MDQ: SIR: 0.68 (95% CI: 0.58–0.79)).

**Table 3:** Standardized incidence ratios for second primaries among female breast cancer survivors, stratified by Indices of Multiple Deprivation quintile.

Cancer site	Female—IMD^a^ quintile 1		Female—IMD quintile 2		Female—IMD quintile 3		Female—IMD quintile 4		Female—IMD quintile 5	
	SIR^b^ (95% CI^c^)	O^d^	SIR (95% CI)	O	SIR (95% CI)	O	SIR (95% CI)	O	SIR (95% CI)	O
All sites combined	1.46 (1.43–1.49)	8051	1.28 (1.26–1.31)	9134	1.23 (1.21–1.26)	10,735	1.20 (1.18–1.23)	11,767	1.16 (1.14–1.18)	12,080
All non-breast sites combined	1.34 (1.30–1.37)	6191	1.14 (1.11–1.16)	6807	1.08 (1.06–1.11)	7955	1.06 (1.04–1.08)	8714	1.00 (0.98–1.02)	8752
Contralateral breast	2.11 (2.01–2.21)	1860	2.06 (1.98–2.15)	2327	2.02 (1.95–2.10)	2780	1.97 (1.90–2.04)	3053	2.00 (1.93–2.07)	3328
Lung	1.86 (1.77–1.95)	1635	1.32 (1.26–1.39)	1513	1.00 (0.95–1.06)	1411	0.85 (0.81–0.90)	1344	0.70 (0.66–0.74)	1180
Colorectum	1.10 (1.03–1.18)	857	1.06 (0.99–1.12)	1062	1.07 (1.01–1.12)	1311	1.11 (1.05–1.17)	1522	1.08 (1.03–1.13)	1570
Endometrium	2.07 (1.92–2.23)	706	1.93 (1.80–2.06)	843	1.94 (1.83–2.07)	1049	1.82 (1.71–1.93)	1108	1.73 (1.63–1.84)	1136
Ovary	1.42 (1.28–1.58)	380	1.21 (1.10–1.34)	415	1.26 (1.16–1.38)	530	1.28 (1.18–1.38)	601	1.16 (1.07–1.26)	587
Melanoma	0.68 (0.58–0.79)	161	0.91 (0.80–1.02)	274	1.11 (1.00–1.22)^e^	408	1.23 (1.12–1.34)	509	1.41 (1.30–1.52)	624
Pancreas	1.31 (1.16–1.48)	267	1.21 (1.08–1.35)	321	1.09 (0.98–1.21)	355	1.17 (1.06–1.28)	424	1.17 (1.06–1.28)	450
Non-Hodgkin's lymphoma	1.03 (0.91–1.17)	250	0.92 (0.81–1.03)	287	0.81 (0.72–0.90)	311	1.00 (0.91–1.10)	431	0.94 (0.85–1.03)	433
Kidney	1.37 (1.20–1.56)	233	1.12 (0.99–1.27)	247	1.18 (1.05–1.31)	318	1.03 (0.92–1.16)	314	0.96 (0.86–1.08)	312
Blood (non-myeloid leukaemia)	1.12 (0.91–1.37)	96	1.02 (0.84–1.22)	113	1.15 (0.98–1.35)	157	1.04 (0.88–1.21)	158	1.05 (0.89–1.22)	169
Blood (myeloid leukaemia)	1.51 (1.22–1.86)	90	1.59 (1.32–1.90)	122	1.62 (1.37–1.90)	152	1.61 (1.38–1.87)	169	1.54 (1.32–1.79)	172
Head and neck	1.38 (1.19–1.60)	179	1.10 (0.94–1.27)	182	1.06 (0.93–1.22)	216	1.12 (0.99–1.26)	255	0.93 (0.81–1.06)	227
Bladder	1.30 (1.11–1.51)	168	1.11 (0.96–1.28)	188	1.10 (0.96–1.25)	226	0.95 (0.82–1.08)	216	0.85 (0.73–0.97)	204
Oesophagus	1.44 (1.24–1.67)	178	1.11 (0.96–1.29)	179	0.98 (0.85–1.13)	193	1.02 (0.89–1.16)	222	0.93 (0.81–1.06)	214
Stomach	1.69 (1.45–1.96)	181	1.38 (1.19–1.59)	192	1.12 (0.97–1.29)	189	1.06 (0.92–1.22)	198	0.95 (0.82–1.10)	187
Blood (myeloma)	0.91 (0.73–1.11)	92	0.81 (0.66–0.97)	106	0.97 (0.83–1.14)	157	1.04 (0.90–1.20)	189	0.85 (0.73–0.99)	164
Liver	1.26 (1.02–1.54)	95	0.94 (0.76–1.15)	92	1.05 (0.87–1.25)	126	0.96 (0.81–1.14)	130	1.09 (0.92–1.27)	156
Brain and central nervous system	0.94 (0.73–1.19)	70	0.77 (0.60–0.96)	73	0.96 (0.79–1.15)	112	0.79 (0.65–0.96)	104	0.92 (0.77–1.10)	130
Thyroid	1.16 (0.89–1.49)	61	1.22 (0.96–1.51)	80	1.33 (1.09–1.61)	106	0.93 (0.74–1.15)	83	1.36 (1.14–1.62)	130

a Indices of Multiple Deprivation.

b Standardized Incidence Ratio.

c Confidence Interval.

d Observed count of second primaries.

e Although the lower/upper confidence interval boundary was rounded to 1.00, the result was significant (p < 0.05).

We saw a difference in female myeloid leukaemia SIRs by receipt of chemotherapy (Chemotherapy: SIR: 2.71 (95% CI: 2.36–3.08). No chemotherapy: SIR: 1.32 (95% CI: 1.20–1.44)). We also estimated endometrial SPC SIRs of 2.30 (95% CI: 2.21–2.40) for females diagnosed with first breast cancer prior to 2010 who received hormonal therapy, 1.26 (95% CI: 1.15–1.39) for females diagnosed with first breast cancer in 2010 or later who received hormonal therapy, 1.95 (95% CI: 1.86–2.04) for females diagnosed with first breast cancer prior to 2010 who did not receive hormonal therapy, and 1.26 (95% CI: 1.15–1.37) for females diagnosed with first breast cancer in 2010 or later who did not receive hormonal therapy.

Male BC survivors were at elevated risks of SPCs at all sites combined (SIR: 1.15 (95% CI: 1.05–1.26)) and all non-breast sites combined (SIR: 1.10 (95% CI: 1.00–1.20), p-value <0.05). Male BC survivors were also at elevated contralateral breast (SIR: 55.4 (95% CI: 35.5–82.4)), prostate (SIR: 1.58 (95% CI: 1.36–1.82)) and thyroid (SIR: 3.74 95% CI: 1.01–9.58)) SPC risks (Table 2). We saw no large differences in non-breast SPC risks by age at BC diagnosis or IMD quintile, although the sample sizes were small (Supplementary Material).

### Incidence rates and cumulative risks

The IRs per 10,000py of non-breast SPCs among females aged under 50 and 50 and over at BC diagnosis peaked at 99.9 (95% CI: 89.2–112) and 149 (95% CI: 138–161) respectively, between 20 and 25 years of follow-up (Table 4). The corresponding 25-year CRs were 15% (95% CI: 14%–16%) and 28% (95% CI: 27%–29%) (Table 4).

**Table 4:** Incidence rates, cumulative risks, and associated statistics for second primary cancer risks.

Age at BC^a^ diagnosis	FU^b^ elapsed	Total py^c^	Number BC	O^d^	IR^e^/10,000 py (95% CI^f^)	Cumulative risk (95% CI)
**Second cancer site: all non-breast (among female BC survivors)**						
Under 50	0-5 y^g^	500,849	127,048	1403	28.0 (26.6–29.5)	1.4% (1.3%–1.5%)
Under 50	5-10 y	284,397	75,638	1113	39.1 (36.9–41.5)	3.4% (3.2%–3.5%)
Under 50	10-15 y	175,633	43,167	982	55.9 (52.5–59.5)	6.1% (5.9%–6.3%)
Under 50	15-20 y	103,490	27,707	766	74.0 (68.9–79.4)	9.5% (9.2%–9.8%)
Under 50	20-25 y	30,424	12,875	304	99.9 (89.2–112)	15% (14%–16%)
50 or over	0-5 y	1,678,273	454,355	15,095	89.9 (88.5–91.4)	4.5% (4.4%–4.5%)
50 or over	5-10 y	832,385	235,309	9285	112 (109–114)	9.7% (9.6%–9.9%)
50 or over	10-15 y	433,064	116,223	5921	137 (133–140)	16% (16%–16%)
50 or over	15-20 y	202,895	61,099	2933	145 (139–150)	22% (21%–22%)
50 or over	20-25 y	44,455	21,178	664	149 (138–161)	28% (27%–29%)
**Second cancer site: all non-breast (among male BC survivors)**						
Under 50	0-5 y	1116	293	5	44.8 (17.0–98.2)	2.3% (0.25%–4.2%)
Under 50	5-10 y	580	161	1	17.2 (1.56–80.3)	3.1% (0.50%–5.6%)
Under 50	10-15 y	352	86	3	85.2 (23.6–227)	7.5% (1.9%–13%)
Under 50	15-20 y	214	55	1	46.6 (4.23–217)	9.5% (2.6%–16%)
Under 50	20-25 y	72	29	0	–	9.5% (2.6%–16%)
50 or over	0-5 y	11,280	3269	267	237 (210–266)	11% (9.7%–12%)
50 or over	5-10 y	4697	1452	111	236 (195–283)	21% (19%–23%)
50 or over	10-15 y	2006	570	62	309 (239–393)	32% (29%–36%)
50 or over	15-20 y	817	260	25	306 (203–444)	43% (38%–47%)
50 or over	20-25 y	132	67	4	303 (101–720)	51% (38%–61%)
**Second cancer site: contralateral breast (among female BC survivors)**						
Under 50	0-5 y	454,222	125,203	1454	32.0 (30.4–33.7)	1.6% (1.5%–1.7%)
Under 50	5-10 y	253,909	67,225	997	39.3 (36.9–41.8)	3.5% (3.4%–3.7%)
Under 50	10-15 y	157,016	38,672	801	51.0 (47.6–54.6)	6.0% (5.7%–6.2%)
Under 50	15-20 y	92,193	24,714	511	55.4 (50.8–60.4)	8.6% (8.3%–8.9%)
Under 50	20-25 y	27,407	11,502	155	56.6 (48.2–66.0)	11% (10%–11%)
50 or over	0-5 y	1,578,741	447,494	3775	23.9 (23.2–24.7)	1.2% (1.2%–1.2%)
50 or over	5-10 y	779,312	219,932	2586	33.2 (31.9–34.5)	2.9% (2.8%–3.0%)
50 or over	10-15 y	405,861	109,023	1911	47.1 (45.0–49.2)	5.2% (5.0%–5.3%)
50 or over	15-20 y	189,854	57,212	928	48.9 (45.8–52.1)	7.4% (7.2%–7.6%)
50 or over	20-25 y	41,968	19,829	230	54.8 (48.1–62.2)	9.9% (9.4%–10%)
**Second cancer site: contralateral breast (among male BC survivors)**						
Under 50	0-5 y	1101	290	1	9.08 (0.82–42.3)	0.39% (0.0%–1.1%)
Under 50	5-10 y	570	158	2	35.1 (7.00–113)	2.6% (0.0%–5.7%)
Under 50	10-15 y	344	84	1	29.1 (2.64–136)	4.2% (0.0%–8.5%)
Under 50	15-20 y	209	54	0	–	4.2% (0.0%–8.5%)
Under 50	20-25 y	70	28	0	–	4.2% (0.0%–8.5%)
50 or over	0-5 y	11,237	3257	8	7.12 (3.36–13.4)	0.33% (0.094%–0.57%)
50 or over	5-10 y	4675	1446	6	12.8 (5.33–26.5)	0.97% (0.39%–1.5%)
50 or over	10-15 y	1988	566	5	25.1 (9.54–55.1)	2.2% (0.94%–3.5%)
50 or over	15-20 y	811	258	1	12.3 (1.12–57.5)	2.8% (1.1%–4.4%)
50 or over	20-25 y	132	67	0	–	2.8% (1.1%–4.4%)
**Second cancer site: endometrium**						
Under 50	0-5 y	481,223	124,111	195	4.05 (3.51–4.65)	0.21% (0.18%–0.24%)
Under 50	5-10 y	264,930	71,212	196	7.40 (6.42–8.49)	0.58% (0.52%–0.64%)
Under 50	10-15 y	162,062	39,935	124	7.65 (6.39–9.09)	0.98% (0.89%–11%)
Under 50	15-20 y	94,947	25,464	87	9.16 (7.39–11.2)	1.4% (1.3%–1.6%)
Under 50	20-25 y	27,826	11,785	28	10.1 (6.83–14.3)	2.0% (1.7%–2.3%)
50 or over	0-5 y	1,617,796	440,211	1676	10.4 (9.87–10.9)	0.54% (0.51%–0.56%)
50 or over	5-10 y	794,676	225,231	1314	16.5 (15.7–17.4)	1.4% (1.3%–1.4%)
50 or over	10-15 y	411,098	110,653	826	20.1 (18.8–21.5)	2.4% (2.3%–2.4%)
50 or over	15-20 y	191,558	57,794	337	17.6 (15.8–19.5)	3.2% (3.1%–3.3%)
50 or over	20-25 y	41,921	19,974	59	14.1 (10.8–18.0)	3.8% (3.5%–4.2%)
**Second cancer site: ovary**						
Under 50	0-5 y	477,350	125,873	165	3.46 (2.96–4.01)	0.18% (0.15%–0.21%)
Under 50	5-10 y	258,687	69,634	116	4.48 (3.72–5.36)	0.40% (0.35%–0.45%)
Under 50	10-15 y	158,414	38,946	96	6.06 (4.94–7.37)	0.71% (0.63%–0.79%)
Under 50	15-20 y	93,671	25,004	70	7.47 (5.87–9.38)	1.1% (0.95%–1.2%)
Under 50	20-25 y	27,826	11,696	21	7.55 (4.81–11.3)	1.4% (1.2%–1.6%)
50 or over	0-5 y	1,638,765	446,105	942	5.75 (5.39–6.12)	0.29% (0.27%–0.31%)
50 or over	5-10 y	806,857	228,452	597	7.40 (6.82–8.01)	0.67% (0.63%–0.70%)
50 or over	10-15 y	418,814	112,518	331	7.90 (7.09–8.79)	1.1% (1.0%–1.1%)
50 or over	15-20 y	195,633	58,982	128	6.54 (5.48–7.75)	1.4% (1.3%–1.5%)
50 or over	20-25 y	42,862	20,408	47	11.0 (8.16–14.4)	1.9% (1.7%–2.1%)

a Breast Cancer.

b Follow up.

c Person-Years.

d Observed count of second primaries.

e Incidence Rate.

f Confidence Interval.

g Years.

The IR of CBC among females peaked at 56.6 (95% CI: 48.2–66.0) and 54.8 (95% CI: 48.1–62.2) in the younger and older age groups respectively between 20 and 25 years of follow-up, and we estimated the corresponding 25-year CRs as 11% (95% CI: 10%–11%) and 9.9% (95% CI: 9.4%–10%). Further site-specific IRs and CRs may be seen in Table 4 and the Supplementary Material.

Among males, IRs of non-breast SPCs respectively peaked at 85.2 (95% CI: 23.6–227) and 309 (95% CI: 239–393) between 10 and 15 years of follow-up among those diagnosed with BC at under age 50 and at age 50 or over. The 25-year CRs were 9.5% (95% CI: 2.6%–16%) and 51% (95% CI: 38–61%).

CBC IRs in males peaked at 25.1 (95% CI: 9.54–55.1) among those first diagnosed with BC at age 50 or over, with the peak reached between 10 and 15 years of follow-up. We estimated the 25-year CR as 2.8% (95% CI: 1.1%–4.4%). There were low numbers of CBCs in males first diagnosed with BC at under age 50. IRs and CRs for the younger age group may be seen in Table 4.

### Associations with socio-demographic factors, tumour characteristics, and treatments

Higher age at BC diagnosis was associated with increasing non-breast SPC risks for both females (HR per year increase: 1.04, 95% CI: 1.04–1.04) and males (HR per year increase: 1.04 (95% CI: 1.03–1.05)) (Table 5). Analyses which treated age at first BC diagnosis as a 5-year categorical variable provided consistent results (Supplementary Material). After 2004, the risk of non-breast SPCs among females increased with more recent BC diagnosis relative to 1995–1999 (2005–09: HR: 1.07 (95% CI: 1.04–1.11), 2010–14: HR: 1.09 (95% CI: 1.05–1.13), 2015–19: HR: 1.10 (95% CI: 1.05–1.15)). In line with the SIR estimates, we found significant evidence for increased non-breast SPC risks among more deprived females, relative to the IMD LDQ (quintile 4: HR: 1.06 (95% CI: 1.03–1.09), quintile 1 (MDQ): HR: 1.35 (95% CI: 1.30–1.39), p-trend: <0.01). However, there were no significant differences in CBC risk between females in different IMD quintiles. Non-breast SPC risks differed by ethnicity, with decreased risks for females of Asian (HR: 0.70 (95% CI: 0.64–0.77)), Black (HR: 0.82 (95% CI: 0.73–0.92)), Chinese (HR: 0.57 (95% CI: 0.42–0.77)), and other non-White, non-mixed (HR: 0.82 (95% CI: 0.71–0.93)) ethnicities relative to females of White recorded ethnicity.

**Table 5:** Associations between socio-demographic, tumour, and treatment characteristics and second primary cancer risk.

Gender:	Female		Female		Female		Female		Male
Second primary cancer site:	All non-breast		Contralateral breast		Endometrium		Ovary		All non-breast
	Adjusted for age at BC^a^ dx^b^ only	Multivariable model	Adjusted for age at BC dx only	Multivariable model	Adjusted for age at BC dx only	Multivariable model	Adjusted for age at BC dx only	Multivariable model	Adjusted for age at BC dx only
	HR^c^ (95% CI^d^)	HR (95% CI)	HR (95% CI)	HR (95% CI)	HR (95% CI)	HR (95% CI)	HR (95% CI)	HR (95% CI)	HR (95% CI)
**Age at first BC diagnosis**^e^									
1y^f^ of additional age	1.04 (1.04–1.04)	1.04 (1.04–1.04)	0.99 (0.99–0.99)	0.99 (0.99–0.99)	1.03 (1.02–1.03)	1.03 (1.02–1.03)	1.01 (1.01–1.02)	1.02 (1.01–1.02)	1.04 (1.03–1.05)
**Year of first BC diagnosis (reference group: 1995**–**1999)**									
2000–2004	1.02 (0.99–1.05)	1.02 (0.99–1.05)	1.09 (1.05–1.14)	1.09 (1.04–1.14)	0.83 (0.77–0.89)	0.83 (0.77–0.89)	1.02 (0.92–1.13)	1.00 (0.91–1.11)	0.84 (0.65–1.09)
2005–2009	1.08 (1.05–1.12)	1.07 (1.04–1.11)	1.08 (1.03–1.14)	1.08 (1.02–1.14)	0.73 (0.67–0.80)	0.73 (0.66–0.79)	0.92 (0.82–1.05)	0.90 (0.80–1.02)	0.93 (0.68–1.25)
2010–2014	1.10 (1.06–1.14)	1.09 (1.05–1.13)	0.94 (0.89–1.00)^k^	0.93 (0.87–0.99)	0.63 (0.57–0.70)	0.63 (0.57–0.70)	0.80 (0.70–0.91)	0.77 (0.67–0.88)	1.11 (0.85–1.45)
2015–2019	1.10 (1.05–1.15)	1.10 (1.05–1.15)	0.91 (0.84–0.98)	0.89 (0.82–0.97)	0.57 (0.50–0.66)	0.57 (0.50–0.66)	0.80 (0.67–0.96)	0.78 (0.65–0.93)	0.97 (0.69–1.37)
**Ethnicity (reference group: White)**									
Asian	0.75 (0.69–0.82)	0.70 (0.64–0.77)	1.00 (0.89–1.13)	1.00 (0.88–1.12)	1.28 (1.06–1.55)	1.32 (1.10–1.60)	0.90 (0.66–1.21)	–	0.52 (0.25–1.11)
Black	0.91 (0.81–1.02)	0.82 (0.73–0.92)	1.04 (0.89–1.22)	1.02 (0.87–1.20)	1.09 (0.82–1.46)	1.11 (0.83–1.48)	0.89 (0.59–1.34)	–	1.68 (0.86–3.28)
Chinese	0.58 (0.43–0.78)	0.57 (0.42–0.77)	0.58 (0.38–0.91)	0.59 (0.38–0.92)	0.51 (0.21–1.22)	0.53 (0.22–1.28)	0.71 (0.26–1.89)	–	1.75 (0.22–14.09)
Mixed	0.90 (0.74–1.10)	0.87 (0.71–1.06)	1.08 (0.81–1.43)	1.08 (0.81–1.43)	0.91 (0.53–1.57)	0.94 (0.55–1.62)	1.51 (0.85–2.66)	–	1.02 (0.25–4.11)
Other	0.83 (0.73–0.95)	0.82 (0.71–0.93)	0.77 (0.63–0.95)	0.78 (0.63–0.96)	0.85 (0.60–1.22)	0.87 (0.61–1.24)	1.01 (0.64–1.58)	–	0.61 (0.15–2.46)
**Indices of Multiple Deprivation quintile (reference group: 5—least deprived)**									
1—most deprived	1.33 (1.29–1.37)	1.35 (1.30–1.39)	1.06 (1.00–1.12)	–	1.19 (1.08–1.30)	1.17 (1.07–1.29)	1.21 (1.07–1.38)	1.21 (1.07–1.38)	0.88 (0.64–1.20)
2	1.13 (1.10–1.17)	1.14 (1.11–1.18)	1.04 (0.99–1.10)	–	1.10 (1.00–1.20)^k^	1.09 (1.00–1.19)	1.04 (0.92–1.18)	1.04 (0.91–1.18)	1.09 (0.83–1.44)
3	1.08 (1.05–1.12)	1.09 (1.06–1.12)	1.03 (0.98–1.08)	–	1.12 (1.03–1.21)	1.11 (1.02–1.21)	1.09 (0.97–1.23)	1.09 (0.97–1.23)	1.03 (0.79–1.34)
4	1.06 (1.03–1.09)	1.06 (1.03–1.09)	0.99 (0.95–1.05)	–	1.05 (0.97–1.14)	1.05 (0.96–1.14)	1.10 (0.98–1.23)	1.10 (0.98–1.23)	1.04 (0.80–1.35)
**Size of first breast tumour (reference group: <2 cm**^g^**)**									
≥2 cm	1.00 (0.98–1.03)	–	1.13 (1.09–1.17)	1.10 (1.05–1.14)	1.00 (0.94–1.07)	–	1.02 (0.93–1.12)	–	1.02 (0.84–1.24)
**Number of nodes involved in first BC (reference group: 0)**									
>0	0.99 (0.96–1.03)	–	1.04 (0.99–1.09)	–	0.97 (0.89–1.06)	–	0.99 (0.89–1.11)	–	1.00 (0.75–1.35)
**Grade of first BC (reference group: 1)**									
2	1.02 (0.99–1.05)	1.02 (0.99–1.05)	1.05 (1.00–1.10)	1.01 (0.96–1.06)	0.95 (0.88–1.02)	–	1.08 (0.96–1.20)	1.08 (0.96–1.21)	1.15 (0.86–1.53)
3	1.09 (1.06–1.12)	1.08 (1.04–1.11)	1.16 (1.10–1.22)	1.05 (0.98–1.12)	0.93 (0.86–1.01)	–	1.49 (1.33–1.67)	1.39 (1.21–1.60)	1.26 (0.92–1.72)
**Morphology of first BC (reference group: Ductal)**									
Lobular	0.97 (0.94–1.00)	0.99 (0.96–1.02)	1.11 (1.05–1.17)	1.13 (1.07–1.20)	0.94 (0.85–1.02)	–	0.91 (0.80–1.03)	**–**	1.76 (0.99–3.13)
Other	0.97 (0.94–0.99)	0.99 (0.96–1.02)	0.97 (0.93–1.02)	0.98 (0.93–1.02)	1.01 (0.93–1.09)	–	0.98 (0.88–1.09)	**–**	1.00 (0.79–1.27)
**ER**^h^**status of first BC (reference group: Negative)**									
Positive	0.92 (0.89–0.95)	0.93 (0.89–0.97)	0.77 (0.73–0.81)	0.80 (0.75–0.86)	1.12 (1.00–1.25)^k^	1.07 (0.95–1.20)	0.70 (0.60–0.82)	0.85 (0.69–1.03)	1.40 (0.21–9.54)
**HER2**^i^**status of first BC (reference group: Negative)**									
Positive	0.92 (0.87–0.97)	0.88 (0.83–0.94)	0.90 (0.83–0.99)	0.85 (0.77–0.93)	0.85 (0.72–1.00)^k^	0.88 (0.74–1.04)	0.95 (0.81–1.11)	–	0.67 (0.36–1.25)
**Has had chemotherapy**^j^**(reference group: Negative)**									
Positive	1.05 (1.02–1.08)	1.02 (0.99–1.05)	1.09 (1.05–1.13)	1.02 (0.98–1.07)	0.93 (0.86–1.00)^k^	1.00 (0.92–1.08)	1.15 (1.04–1.26)	1.01 (0.91–1.12)	1.23 (0.96–1.57)
**Has had radiotherapy**^j^**(reference group: Negative)**									
Positive	1.08 (1.06–1.10)	1.07 (1.05–1.10)	1.01 (0.98–1.05)	1.04 (1.00–1.08)^k^	1.07 (1.01–1.13)	1.10 (1.03–1.16)	1.02 (0.95–1.11)	1.06 (0.97–1.15)	1.00 (0.84–1.20)
**Has had hormonal therapy**^j^**(reference group: Negative)**									
Positive	1.02 (1.00–1.04)^k^	1.02 (1.00–1.04)	0.88 (0.85–0.91)	0.90 (0.87–0.94)	1.14 (1.08–1.21)	1.09 (1.02–1.16)	0.88 (0.81–0.95)	0.92 (0.84–1.01)	0.94 (0.78–1.12)

**Note:** Each multivariable Cox model was adjusted for all variables found significant in the initial set of Cox models adjusted only for age, other than the models fit to assess the effect of IMD quintile, HER2 status, and radiotherapy status on the risks of all non-breast SPCs combined in females and the risks of endometrial SPCs. This is because HER2 status was not significantly correlated with IMD quintile or radiotherapy status in the cohorts used to fit these models. Therefore, when assessing the effect of radiotherapy or IMD quintile on non-breast and endometrial SPC risks, HER2 status was dropped from the corresponding Cox models. When assessing the effect of HER2 status on non-breast and endometrial SPC risks, IMD quintile was dropped from the corresponding Cox models. However, due to the inclusion of chemotherapy and hormonal therapy status, radiotherapy status was retained in the models fit to assess the effect of HER2 status on non-breast and endometrial SPC risks, in order to account for tandem treatment effects (Methods section). Following inspection of the log(-log) plots, no variable was judged to violate the proportional hazards assumption, so no variables were dropped from the Cox models for this reason.

a Breast Cancer.

b Diagnosis/Diagnosed.

c Hazard Ratio.

d Confidence Interval.

e In this section, the results headed ‘Adjusted for age at BC dx only’ are from a univariate Cox model that included only age at BC diagnosis as a predictor.

f Year.

g Centimetres.

h Estrogen Receptor.

i Human Epidermal growth factor Receptor 2.

j By start of follow-up.

k Although the lower/upper confidence interval boundary was rounded to 1.00, the result was significant (p < 0.05).

Female BC survivors diagnosed with grade 3, rather than grade 1, first BC were at increased non-breast SPC risks (HR: 1.08 (95% CI: 1.04–1.11)), whereas non-breast SPC risks were lower for those whose first BC was ER-positive rather than ER-negative (HR: 0.93 (95% CI: 0.89–0.97)) or HER2-positive rather than HER2-negative (HR: 0.88 (95% CI: 0.83–0.94)). Radiotherapy was associated with a 7% increase (95% CI: 5–10%) in non-breast SPC risks.

There was significant evidence that increasing age at BC diagnosis was associated with decreased CBC risk (HR per year increase: 0.99 (95% CI: 0.99–0.99)), and with increased endometrial (HR per year increase: 1.03 (95% CI: 1.02–1.03)) and ovarian (HR per year increase: 1.02 (95% CI: 1.01–1.02)) SPC risks. Radiotherapy was associated with increased CBC risk (HR: 1.04 (95% CI: 1.00–1.08, p < 0.05)), whereas hormonal therapy was associated with a decreased risk (HR: 0.90 (95% CI: 0.87–0.94)). There was significant evidence that radiotherapy (HR: 1.10 (95% CI: 1.03–1.16)) and hormonal therapy (HR: 1.09 (95% CI: 1.02–1.16)) increased the risk of endometrial SPCs.

HR estimates for the associations of socio-demographic factors and tumour characteristics with SPC risks may be seen in Table 5 and the Supplementary Material.

## Discussion

In this study we used population-scale linked cancer registration data to estimate SPC risks following male and female BC in England and assess how these risks varied by socio-demographic factors, breast tumour characteristics and treatments. This is the largest published cohort study of its kind to date, with a longer mean follow-up than any other study of comparable size.^5^^,^^6^

There were significantly increased CBC and non-breast SPC risks for BC survivors of either gender compared to population-level risks. The greatest SIRs were observed for second cancer of the contralateral breast, and for endometrium and prostate cancer in females and males, respectively. Non-breast SPC risks were higher for females diagnosed with BC before age 50 and for those from more deprived regions, compared to population-level non-breast cancer risks. We also found that non-breast SPC risks differed by the grade of the first primary, and that breast cancer survivors diagnosed after 2004, who received radiotherapy, or were from more deprived regions were at elevated non-breast SPC risks compared to BC survivors diagnosed from 1995 to 1999, who did not receive radiotherapy, and who were from the IMD LDQ. Non-breast SPC risks were lower for female BC survivors of Asian, Black, Chinese, or other non-White, non-mixed ethnicities compared to white ethnicity female BC survivors and for females whose first BC was ER-positive rather than ER-negative and HER2-positive rather than HER2-negative.

To our knowledge, no previous study of SPC risks following male BC has accounted for any of these factors other than age at, and year of, BC diagnosis. No study has accounted for socioeconomic deprivation in female BC survivors, and no population-based study has estimated associations between tumour size, lymph node involvement, grade or morphology and SPC risks following BC in females. Only one small study has accounted for treatment with hormonal therapy^15^ at combined sites, without examining treatment effects on site-specific risks. The current study therefore has provided evidence on possible causes of heterogeneity among previously published SPC risk estimates.^5^^,^^6^ A further strength is that we were able to account for prophylactic and curative surgeries such as partial or full resections at the contralateral breast, ovary, and endometrium in our censoring process when estimating SPC risks at these sites, minimising confounding biases in our risk estimates.^16^

Although our estimated SIR for non-breast SPCs in females is lower than the pooled estimate of 1.24 (95% CI: 1.14–1.36) from a recent meta-analysis,^5^ our findings of elevated SIRs for all non-breast SPCs combined, particularly at the endometrium, are consistent with previous research.^5^^,^^9^ These findings suggest that BC survivors may benefit from enhanced surveillance for specific SPC development, but should be taken in context with our 25-year CR estimates, which were notably higher for CBC than endometrial SPCs. Any specific surveillance recommendations would require separate cost-benefit analyses.

We found higher SIRs for females diagnosed with BC at under age 50, which is consistent with the recent meta-analysis.^5^ A partial explanation could be the higher proportion of pathogenic variants in BC susceptibility genes among younger BC survivors.^17^ Women with germline *BRCA1/2* pathogenic variants are at elevated CBC,^18^ ovarian,^18^^,^^19^ pancreatic,^19^ and stomach^19^ cancer risks and these were the cancers with the biggest differences in SIRs by age in the present study. It should be noted that the 25-year non-breast cumulative SPC risks were higher in the older age group, suggesting that although younger BC survivors may benefit more from enhanced cancer surveillance relative to their age group, clinicians should remain vigilant of SPC risks in the older group. Pathogenic genetic variation may also explain the very high CBC SIR observed in male BC survivors, since *BRCA2* PVs are associated with a high BC risk in males.^19^ In addition, although we did not find significant associations between chemotherapy receipt and SPC risks at the contralateral breast or at all non-breast sites combined, we did observe a clear elevation in myeloid leukaemia SPC SIRs in females treated with chemotherapy compared to those that were not. It is therefore possible that the increased myeloid leukaemia SIRs in females diagnosed with BC at under age 50 compared to those aged 50 or over are partly attributable to increased chemotherapy usage in the younger age group.

We found increased non-breast SPC SIRs in BC survivors diagnosed in more deprived regions, particularly for lung, kidney, head and neck, bladder, oesophagus and stomach SPCs. These may be explained by higher prevalences of smoking, obesity, and alcohol consumption among more deprived groups,^20^ as these are established risk factors for these cancers.^21^, ^22^, ^23^ There was no trend for increasing CBC risks with increasing deprivation, which is consistent with population-level BC risks.^24^ A notable exception was melanoma, for which SPC risks declined with increasing deprivation. This is also consistent with population-level risks.^24^ The significant decreases in non-breast SPC risks for females of Asian, Black, Chinese, and other non-White, non-mixed ethnicities compared to those of White ethnicity are also consistent with population-level risk differences,^25^ and may be partly explained by lower prevalences of overweight and obesity,^26^ lower alcohol consumption,^26^ and lower screening rates^27^ in non-White ethnicity groups.

There was a pattern of increasing non-breast SPC risks for females with more recent BC diagnosis, which is broadly consistent with the IR rise for all cancers combined in the UK over the study period.^11^ However, there were respective decreasing CBC and endometrial SPC risks with later BC diagnoses after 2009 and throughout the study period, in contrast to the population-level risks.^11^ This may be attributable to shorter follow-up times contributed by BC survivors diagnosed later, as CBC risks may increase with follow-up time^9^ and the known elevation in endometrial SPC risk after tamoxifen treatment for BC increases further with increasing time since the treatment.^28^ The lower endometrial cancer risks for those diagnosed with BC after 2009 may also be partly due to an increase in aromatase inhibitor usage in BC treatment in later years, which is associated with a lower endometrial SPC risk than tamoxifen^29^ but a similar decrease in risk was observed in our data among those did not receive hormonal therapy.

There was a significant decrease in CBC risk for females treated with hormonal therapy together with significant increases in non-breast SPC risks for females treated with radiotherapy, which are consistent with previous studies.^30^^,^^31^ We also found that BC survivors diagnosed with a first BC 2 cm or greater in size, of lobular morphology, of negative ER status, or of negative HER2 status were at significantly elevated CBC risk.

Any findings should be taken in context with the limitations of our study. Firstly, we cannot rule out surveillance bias,^9^ although starting our follow-up at one year following the first BC diagnosis should have reduced this.^9^ Secondly, data on surgeries were based on coding practices reliant on discharge summaries, which vary in accuracy and completeness between hospitals.^10^ Furthermore, some surgeries which we treated as censoring events for a given SPC will not have entirely removed the risk of that SPC, such as subtotal hysterectomies for endometrial SPCs. Therefore, a small number of SPCs diagnosed at least one year after relevant surgeries (Supplementary Material) were not considered in our risk estimates. We were unable to account for obesity,^23^ smoking status,^21^ alcohol intake,^22^ family history of cancer,^18^ or germline cancer susceptibility^18^^,^^19^ due to a lack of data on these variables. In addition, although we excluded patients with no staging records and confirmed metastatic or non-invasive BC at diagnosis from the cohort, some BC survivors with inconclusive staging data may in fact have had metastatic or non-invasive disease. It should be noted that BC survivors diagnosed in 2013 in the NCRD dataset with missing staging data were found to be older at first breast cancer diagnosis, have higher rates of short-term mortality, be less likely to have received a surgery, be diagnosed with first BC in more socioeconomically deprived regions, present with BC as emergency cases, have higher numbers of comorbidities, and have more severe comorbidities.^32^ We directly adjusted for age at first BC diagnosis in all our SPC risk estimates, stratified or adjusted for year at BC diagnosis and socioeconomic deprivation in our SIR and HR SPC estimates, censored at contralateral breast surgeries, and minimized the influence of the differences in short-term mortality and comorbidities by excluding patients contributing less than one year of follow-up. It is therefore unlikely that the SPC risk estimates would be substantially impacted, but this should be considered when interpreting our results.

Finally, despite the large cohort size and long-term follow-up, some analyses were based on small subgroups of BC survivors or low SPC counts, particularly at rarer cancer sites and in males.

In conclusion, we generated precise relative and absolute SPC risk estimates at combined and specific sites based on comprehensive EHR data, meeting our primary objective. We assessed the variation in SPC risks by a wide range of sociodemographic factors, first tumour characteristics, and BC treatments and found that SPC risks are elevated among BC survivors living in more socioeconomically deprived regions at diagnosis.

These results should facilitate an evidence-based approach to SPC risk management following BC and suggest policy measures to reduce inequalities in smoking and other deprivation-associated cancer risk factors would lessen SPC risks. However, we could not explicitly confirm this due to a lack of smoking, obesity, and alcohol intake data, demonstrating the need for future studies investigating how deprivation-associated factors affect SPC risks. There is also a need for larger studies of SPC risks in non-White ethnicity BC survivors, with less than 14% of this cohort being of non-White ethnicity. Finally, larger studies of SPC risks following male BC are necessary.

## Contributors

IA conducted the statistical analyses and wrote the manuscript. TR, AB, CK, and SJ collected and directly verified the data. HH developed the imputation approach. MT, PP and AA supervised the project and reviewed and edited the manuscript drafts. All authors provided critical feedback to inform the research and analysis.

## Data sharing statement

This work uses data that has been provided by patients and collected by the NHS as part of their care and support. The data are collated, maintained and quality assured by the National Disease Registration Service (NDRS), which is part of NHS England. Data in this manuscript may be accessed through application to NHS England. The plots used to assess the proportional hazards assumption in the Cox models can be made available upon request.

## Declaration of interests

DE is co-investigator on a research grant awarded by AstraZeneca. ACA is listed as creator of the BOADICEA algorithm which has been licensed by Cambridge Enterprise (University of Cambridge). CH receives funding for a clinical fellowship from the Wellcome Trust. BT has received grants from NHS Cancer Programme/SBRI Healthcare and has received personal payments for lecturing at University College London. CT has received honoraria for educational events and scientific participation in a biobanking project (all donated in whole to charity) from Roche and Astrazeneca. IA, HH, and LL receive studentships funded by Cancer Research UK. BT acknowledges payments to her institution from Cancer Research UK. EM notes that Cancer Research UK have funded this program of work. PP acknowledges grant funding from Cancer Research UK received by the University of Cambridge.
