# Supplementary material for: Risks of second primary cancers among 584,965 female and male breast cancer survivors in England: a 25-year retrospective cohort study
Source: Lancet Reg Health Eur. 2024 Apr 24;40:100903. doi: 10.1016/j.lanepe.2024.100903 (PMC11092881; doi:10.1016/j.lanepe.2024.100903)
Supplement: Supplementary Material [file mmc1.docx]

# Supplementary material

# Methods

## Data sources

The NCRD dataset is curated by the National Cancer Registration and Analysis Service (NCRAS), which is part of the National Health Service England, and has collected population-based, individual-level data on those diagnosed with cancer or premalignant conditions in England since 1971^7^. Consent from subjects is not required due to Section 254 of the Health and Social Care Act 2012. Each year NCRAS receives about 25 million records relevant to the NCRD dataset, for around 300,000 diagnosed malignant tumours. Patients are identified primarily by the NHS number (a unique identifier across the English healthcare system), although dates of birth, full names and addresses are also used for identification and data linkage. All new tumour registrations are processed and reviewed by cancer registration officers as a quality control measure, in addition to further automated quality control practices.

The HES APC dataset contains data on hospital of admission, admission dates, discharge dates, diagnoses and procedures occurring during the hospital stay, demographic information, and numerous other variables^10^. Data have been collected since 1989, although the HESID (an identifying variable corresponding to a given hospital episode, allowing the longitudinal tracking of such episodes for a given patient) was first added in 1997. Data are entered by clinical coders and undergo quality control performed by NHSE, who also allocate the HESID. The HES APC dataset was linked to the HES OP dataset to collect information on breast surgery laterality.

We checked the data for errors in chronology, such as death and embarkation dates recorded before BC diagnosis dates, or SPC diagnosis dates recorded after death dates. Data were also checked for further inconsistencies, such as records of surgeries inconsistent with recorded gender, or of morphologies inconsistent with invasive BC. Finally, the data were checked for missing information in variables additional to those mentioned above, such as in date of birth or gender. Depending on the variables and the types of errors observed, records were either filtered from the cohort or assigned sensible default values in place of the erroneous information.

## Details of imputation model:

We imputed missing data using the random forest method with 10 cycles for 10 imputations, separately by gender. We imputed missing data for the following variables:

- Ethnicity (8.9% missing in females, 9.5% missing in males)
- First breast tumour size in centimetres (23.6% missing in females, 20.2% missing in males)
- Number of nodes involved in first breast tumour (40.5% missing in females, 36.6% missing in males)
- Grade of first breast tumour (7.7% missing in females, 9.1% missing in males)
- Morphology of first breast tumour (<0.1% missing in females, complete in males)
- ER status of first breast tumour (51.4% missing in females, 46.3% missing in males)
- HER2 status of first breast tumour (55.3% missing in females, 54.8% missing in males)

We used the following variables as predictors for the missingness mechanism in the imputation model:

- Year of first BC diagnosis
- Age at first BC diagnosis
- Ethnicity
- IMD quintile
- First breast tumour size in centimetres
- Number of nodes involved in first breast tumour
- Grade of first breast tumour
- Morphology of first breast tumour
- ER status of first breast tumour
- HER2 status of first breast tumour
- Vital status (whether patient lived, died, or embarked the UK during follow-up)
- Whether the patient had chemotherapy by the start of follow-up
- Whether the patient had radiotherapy by the start of follow-up
- Whether the patient had hormonal therapy by the start of follow-up
- Whether the patient developed a SPC during follow-up (without censoring at surgeries)^S1^
- A Nelson-Aalen estimator for the development of a SPC (without censoring at surgeries)^S1^

## ICD-10 code groupings used for cancer sites:

We defined cancer sites using the ICD-10 code groups used by Cancer Research UK^11^, which may be seen in table S1.

**Table S1: ICD-10 codes used to define cancer sites**

| **Cancer site** | **ICD-10 code group** |
| --- | --- |
| All sites combined | C00-C97, excluding C44 |
| All non-breast sites combined | C00-C97, excluding C44 and C50 |
| Contralateral breast | C50 |
| Lung | C33-34 |
| Colorectum | C18-20 |
| Endometrium | C54-55 |
| Ovary | C56-57.4 |
| Melanoma | C43 |
| Pancreas | C25 |
| Non-Hodgkin’s lymphoma | C82-86 |
| Kidney | C64-66, C68 |
| Blood (non-myeloid leukaemia) | C91-95, excluding C92.0, C92.1, C92.4, C92.5, C92.6, C92.8, C93.0, C94.0, C94.2 |
| Blood (myeloid leukaemia) | C92.0, C92.1, C92.4, C92.5, C92.6, C92.8, C93.0, C94.0, C94.2 |
| Head and neck | C00-14, C30-32 |
| Bladder | C67 |
| Oesophagus | C15 |
| Stomach | C16 |
| Blood (myeloma) | C90 |
| Liver | C22 |
| Brain and central nervous system | C70-72 |
| Thyroid | C73 |
| Prostate | C61 |

## Details of surgical censoring:

Follow-up for second ovarian or endometrial cancers was censored at one year following any surgery outlined in table S2. Follow-up for CBC was also censored at one year following any surgery outlined in table S2 unless the laterality of the surgery was unknown. In this case, the surgery was taken as a censoring event for CBC only if it occurred before the first BC diagnosis or at least one year following the first BC diagnosis, as any surgery performed within one year of the first BC diagnosis was assumed to have been performed on the ipsilateral, cancerous breast. The surgeries in Table S2 are a subset of those defined as breast cancer curative within the HES APC dataset and were chosen to capture common surgeries administered to BC patients whilst restricting the number of surgical procedures extracted to a practical number.

Some of our analyses assessed the risks of second primaries at combined sites, including both sites mentioned above and sites which we had no surgical data on. Our censoring approaches for these analyses are described here.

When calculating SIRs for SPCs at all sites and at all non-breast sites combined, we calculated separate SIRs for contralateral breast, ovarian, and endometrial SPCs whilst censoring at one year following relevant surgeries and combined these with a SIR for SPCs at all other sites combined in which we did not censor at any surgery.

When calculating IRs and CRs for SPCs at combined sites, we did not censor at surgeries. We did censor at relevant surgeries (Table S1) when estimating IRs and CRs of contralateral breast, endometrial, and ovarian SPCs.

When calculating HRs to assess associations with SPC risks at combined sites between BC survivors, we did not treat surgeries as censoring events. We did treat surgeries as censoring events when calculating HRs to assess associations with contralateral breast, endometrial, and ovarian SPC risks.

**Table S2: Censoring surgeries for contralateral breast, ovarian, and endometrial second primaries**

| **Censoring surgeries for second primary contralateral breast cancer** | **Censoring surgeries for second primary endometrial cancer** | **Censoring surgeries for second primary ovarian cancer** |
| --- | --- | --- |
| Total mastectomy and excision of both pectoral muscles and part of chest wall | Abdominal hysterocolpectomy and excision of periuterine tissue | Abdominal hysterocolpectomy and excision of periuterine tissue |
| Total mastectomy and excision of both pectoral muscles NEC^1^ | Abdominal hysterectomy and excision of periuterine tissue NEC | Abdominal hysterectomy and excision of periuterine tissue NEC |
| Total mastectomy and excision of pectoralis minor muscle | Abdominal hysterocolpectomy NEC | Vaginal hysterocolpectomy and excision of periuterine tissue |
| Total mastectomy NEC | Total abdominal hysterectomy NEC | Bilateral salpingoophorectomy |
| Subcutaneous mastectomy | Subtotal abdominal hysterectomy | Bilateral oophorectomy NEC |
| Skin sparing mastectomy | Other specified abdominal excision of uterus | Salpingoophorectomy of remaining solitary fallopian tube and ovary |
| Other specified total excision of breast | Unspecified abdominal excision of uterus | Oophorectomy of remaining solitary ovary NEC |
| Unspecified total excision of breast | Vaginal hysterocolpectomy and excision of periuterine tissue | Other specified unilateral excision of adnexa of uterus |
| Quadrantectomy of breast | Vaginal hysterectomy and excision of periuterine tissue NEC | Unspecified unilateral excision of adnexa of uterus |
| Partial excision of breast NEC | Vaginal hysterocolpectomy NEC | Salpingoophorectomy NEC |
| Excision of lesion of breast NEC | Other specified vaginal excision of uterus | Oophorectomy NEC |
| Re-excision of breast margins | Unspecified vaginal excision of uterus | Other specified other open operations on ovary |
| Wire guided partial excision of breast | Total exenteration of pelvis | Total exenteration of pelvis |
| Wire guided excision of lesion of breast | Anterior exenteration of pelvis | Anterior exenteration of pelvis |
| Other specified other excision of breast | Posterior exenteration of pelvis | Posterior exenteration of pelvis |
| Unspecified other excision of breast | Other specified clearance of pelvis | Other specified clearance of pelvis |
| Subareolar excision of mammary duct | Unspecified clearance of pelvis | Unspecified clearance of pelvis |
| Excision of mammary duct NEC | .. |  |
| Excision of lesion of mammary duct | .. | .. |
| Excision of nipple | .. | .. |
| Extirpation of lesion of nipple | .. | .. |
| Capsulectomy of breast | .. | .. |
| Interstitial laser destruction of lesion of breast | .. | .. |
| Other specified destruction of lesion of breast | .. | .. |
| Unspecified destruction of lesion of breast | .. | .. |

1: Not Elsewhere Specified

# R packages used

We used the packages data.table^S2^, DBI^S3^, dplyr^S4^, epiR^S5^, forcats^S6^, getPass^S7^, mice^S8^, lubridate^S9^, readxl^S10^, rJava^S11^, RJDBC^S12^, survival^S13^, survminer^S14^ and svDialogs^S15^ in our statistical analyses, which were all performed in R version 4.2.1^S16^.

# Results

## Further description of the cohort – pathology of the first breast cancer

There was a high degree of missingness in the pathology data for the first breast cancer, with over 50% of data on ER status and HER2 status missing. Of the patients without missing data, the majority were diagnosed with a first breast tumour of less than 2 centimetres in size (39.9%, 23.6% missing data), with no nodes involved (39.5%, 40.5% missing data), of grade 2 (46.8%, 7.7% missing data), of ductal morphology (74.5%, <0.1% missing data), ER-positive (41.4%, 51.4% missing data), and HER2-negative (38.2%, 55.3% data missing). Further details of the pathology of the first BC may be seen in Table S3. A comparison of the distributions of ER and HER2 status between imputed data and complete-case data can be seen in Table S4.

**Table S3: Further cohort description: pathology of first breast cancer**

|  | **Female cohort (all)** | | **Female cohort (with a SPC^1^)** | | **Male cohort (all)** | | **Male cohort (with a SPC)** | |
| --- | --- | --- | --- | --- | --- | --- | --- | --- |
|  | **Number BC^2^ (%)** | **Total FU (py^3^) (%)** | **Number BC (%)** | **Total FU (py) (%)** | **Number BC (%)** | **Total FU (py) (%)** | **Number BC (%)** | **Total FU (py) (%)** |
| **Size of first breast tumour** | | | | | | | | |
| *<2cm^4^* | 231719 (39.9) | 1822537 (42.5) | 21957 (41.7) | 166130 (42.0) | 1292 (36.3) | 8301 (39.0) | 189 (37.5) | 1086 (37.8) |
| *>=2cm* | 212524 (36.6) | 1395526 (32.6) | 17549 (33.4) | 119773 (30.2) | 1551 (43.5) | 8263 (38.9) | 204 (40.5) | 1017 (35.4) |
| *Data missing* | 137160 (23.6) | 1067801 (24.9) | 13114 (24.9) | 110116 (27.8) | 719 (20.2) | 4704 (22.1) | 111 (22.0) | 772 (26.8) |
| **Number of nodes involved in first BC** | | | | | | | | |
| *0* | 229804 (39.5) | 1506795 (35.2) | 17973 (34.2) | 113854 (28.7) | 1230 (34.5) | 6771 (31.8) | 161 (31.9) | 734 (25.5) |
| *>0* | 115842 (19.9) | 752726 (17.6) | 8562 (16.3) | 58302 (14.7) | 1030 (28.9) | 5149 (24.2) | 126 (25.0) | 610 (21.2) |
| *Data missing* | 235757 (40.5) | 2026294 (47.3) | 26085 (49.6) | 223862 (56.5) | 1302 (36.6) | 9348 (44.0) | 217 (43.1) | 1531 (53.2) |
| **Grade of first BC** | | | | | | | | |
| *1* | 95267 (16.4) | 830143 (19.4) | 10232 (19.4) | 83608 (21.1) | 379 (10.6) | 2734 (12.9) | 56 (11.1) | 379 (13.2) |
| *2* | 271961 (46.8) | 1909733 (44.6) | 23321 (44.3) | 167875 (42.4) | 1806 (50.7) | 10216 (48.0) | 239 (47.4) | 1296 (45.1) |
| *3* | 169234 (29.1) | 1148699 (26.9) | 14114 (26.8) | 99651 (25.2) | 1053 (29.6) | 5622 (26.6) | 143 (28.4) | 728 (25.3) |
| *Data missing* | 44941 (7.7) | 397288 (9.2) | 4953 (9.4) | 44886 (11.3) | 324 (9.1) | 2697 (12.6) | 66 (13.1) | 473 (16.5) |
| **Morphology of first BC** | | | | | | | | |
| *Ductal* | 433383 (74.5) | 3143510 (73.3) | 38031 (72.3) | 283841 (71.7) | 2985 (83.8) | 17418 (81.9) | 404 (80.2) | 2251 (78.3) |
| *Lobular* | 67830 (11.7) | 493295 (11.5) | 6283 (11.9) | 46302 (11.7) | 51 (1.4) | 285 (1.3) | 12 (2.4) | 51 (1.8) |
| *Other* | 80161 (13.8) | 648612 (15.1) | 8304 (15.8) | 65868 (16.6) | 526 (14.8) | 3564 (16.8) | 88 (17.5) | 574 (20.0) |
| *Data missing* | 29 (<0.1) | 447 (<0.1) | 2 (<0.1) | 9 (<0.1) | 0 (0) | 0 (0) | 0 (0) | 0 (0) |
| **ER^5^ status of first BC** | | | | | | | | |
| *Negative* | 41906 (7.2) | 196338 (4.6) | 2582 (4.9) | 11366 (2.9) | 26 (0.7) | 70 (0.3) | 1 (0.2) | 8 (0.3) |
| *Positive* | 240825 (41.4) | 1232551 (28.8) | 13343 (25.4) | 62848 (15.9) | 1886 (52.9) | 8159 (40.1) | 200 (39.7) | 659 (22.9) |
| *Data missing* | 298672 (51.4) | 2856975 (66.7) | 36695 (69.7) | 321806 (81.3) | 1650 (46.3) | 12697 (59.6) | 303 (60.1) | 2209 (76.8) |
| **HER2^6^ status of first BC** | | | | | | | | |
| *Negative* | 222360 (38.2) | 983526 (22.9) | 10793 (20.5) | 39051 (9.9) | 1472 (41.3) | 6078 (28.6) | 137 (27.2) | 402 (14.0) |
| *Positive* | 37623 (6.5) | 168388 (3.9) | 1558 (3.0) | 5704 (1.4) | 138 (3.9) | 584 (2.7) | 6 (1.2) | 16 (0.6) |
| *Data missing* | 321420 (55.3) | 3133950 (73.1) | 40269 (76.5) | 351265 (88.7) | 1952 (54.8) | 14606 (68.7) | 361 (71.6) | 2457 (85.5) |

1: Second Primary Cancer, 2: Breast Cancer, 3: Person Years, 4: Centimetres, 5: Estrogen Receptor, 6: Human Epidermal growth factor Receptor 2

**Table S4: Description of ER status and HER2 status of first breast tumour in complete and imputed data**

|  | | **Female cohort (all)** | | **Female cohort (with a SPC^1^)** | | **Male cohort (all)** | | **Male cohort (with a SPC)** | |
| --- | --- | --- | --- | --- | --- | --- | --- | --- | --- |
|  | | **Number BC^2^ (%) – complete data** | **Number BC (%) – imputed data** | **Number BC (%) – complete data** | **Number BC (%) – imputed data** | **Number BC (%) – complete data** | **Number BC (%) – imputed data** | **Number BC (%) – complete data** | **Number BC (%) – imputed data** |
| **ER^3^ status of first BC** | | | | | | | | |  |
| *Negative* | 41906 (14.8) | 461646 (15.5) | 2582 (16.2) | 52734 (14.4) | 26 (1.4) | 54 (0.3) | 1 (0.5) | 11 (0.4) |  |
| *Positive* | 240825 (85.2) | 2525074 (84.5) | 13343 (83.8) | 314216 (85.6) | 1886 (98.6) | 16446 (99.7) | 200 (99.5) | 3019 (99.6) |  |
| **HER2^4^ status of first BC** | | | | | | | | |  |
| *Negative* | 222360 (85.5) | 2804606 (87.3) | 10793 (87.4) | 358407 (89.0) | 1472 (91.4) | 18644 (95.5) | 137 (95.8) | 3487 (96.6) |  |
| *Positive* | 37623 (14.5) | 409594 (12.7) | 1558 (12.6) | 44283 (11.0) | 138 (8.6) | 876 (4.5) | 6 (4.2) | 123 (3.4) |  |

1: Second Primary Cancer, 2: Breast Cancer, 3: Estrogen Receptor, 4: Human Epidermal growth factor Receptor 2

Note: The figures for the imputed data include data from all ten imputed datasets.

## Further descriptions of standardized incidence ratios, incidence rates, cumulative risks, and associations with socio-demographic factors, tumour characteristics, and treatments administered

### Stratified standardized incidence ratios for male BC survivors

We estimated the SIR for non-breast SPCs as 0.91 (95%CI:0.44-1.67) for males aged under 50 at BC diagnosis, and as 1.10 (95%CI:1.00-1.21, p<0.05) for males aged 50 or over at BC diagnosis. We estimated the SIR for non-breast SPCs among the least and most deprived males to respectively be 1.09 (95%CI:0.90-1.30) and 0.94 (95%CI:0.72-1.21).

### Incidence rates and cumulative risks

The IRs per 10,000py for second contralateral breast, endometrial, and ovarian primaries all rose throughout the follow-up period and respectively peaked at 56.6 (95%CI:48.2-66.0), 10.1 (95%CI:6.83-14.3), and 7.55 (95%CI:4.81-11.3) at between 20 and 25 years of follow-up for females diagnosed with BC at under age 50 (Table 4).

The IRs for contralateral breast and ovarian SPCs among those diagnosed with first BC at age 50 or over also peaked between 20 and 25 years of follow-up, with the CBC IR peaking at 54.8 (95%CI:48.1-62.2) and the ovarian SPC IR peaking at 11.0 (95%CI:8.16-15.4). Finally, the IR for second endometrial primaries peaked at 20.1 (95%CI:18.8-21.5) between 10 and 15 years of follow-up among BC survivors aged 50 or over at their first BC diagnosis.

We estimated the 25-year CR of contralateral breast, endometrial, and ovarian SPCs to be 11% (95%CI:10%-11%), 2.0% (95%CI:1.7%-2.3%) and 1.4% (95%CI:1.2%-1.6%) for females first diagnosed with BC at under age 50 and as 9.9% (95%CI:9.4%-10%), 3.8% (95%CI:3.5%-4.2%) and 1.9% (95%CI:1.7%-2.1%) for females first diagnosed with BC at age 50 or over.

A visual representation of the 25-year CRs of non-breast, contralateral breast, endometrial and ovarian SPCs may be seen in Figure S1.

### Associations with socioeconomic factors, tumour characteristics, and treatments administered

#### Contralateral BC

We found significant evidence that increasing age at BC diagnosis was associated with decreasing CBC risk (HR per year increase: 0.99, 95%CI: 0.99-0.99) (Table 5). We also found that CBC risk increased with a later first BC diagnosis until 2009 (2000-04: HR: 1.09 (95%CI:1.04-1.14), 2005-09: HR: 1.08 (95%CI:1.02-1.14), relative to 1995-99). At this point, CBC risks decreased with later diagnosis period (2010-14: HR: 0.93 (95%CI:0.87-0.99), 2015-19: HR: 0.89 (95%CI:0.82-0.97)). We also saw significant evidence that females of Chinese ethnicity (HR: 0.59 (95%CI:0.38-0.92)) and other non-White, non-Mixed ethnicities (HR: 0.78 (95%CI:0.63-0.96)) were at lower CBC risk than those of White ethnicity.

We found significant increases in CBC risks among BC survivors whose first breast tumour was at least 2cm in size (HR: 1.10 (95%CI:1.05-1.14)) or had a lobular morphology (HR: 1.13 (95%CI:1.07-1.20)), whereas those whose first breast tumour was ER-positive (HR: 0.80 (95%CI:0.75-0.86)) or HER2-positive (HR: 0.85 (95%CI:0.77-0.93)) were at lower risk.

#### Endometrial SPCs

We found older BC survivors (HR per year increase: 1.03 (95%CI:1.02-1.03)) to be at significantly increased risk of endometrial SPCs (Table 5). In contrast to the pattern observed for SPC risks at all non-breast sites combined, we saw endometrial SPC risks decrease with a later period of BC diagnosis relative to 1995-1999 (2000-04: HR: 0.83 (95%CI:0.77-0.89), 2005-09: HR: 0.73 (95%CI:0.66-0.79), 2010-14: HR: 0.63 (95%CI:0.57-0.70), 2015-19: HR: 0.57 (95%CI:0.50-0.66)).

We also found BC survivors of Asian ethnicity, from the IMD MDQ, or from IMD quintile 3 to be at significantly elevated risk of endometrial SPCs compared to those of White ethnicity or from the IMD LDQ (Asian ethnicity: HR: 1.32 (95%CI:1.10-1.60), IMD quintile 3: HR: 1.11 (95%CI:1.02-1.21), IMD MDQ: HR: 1.17 (95%CI:1.07-1.29)).

#### Ovarian SPCs

We found increasing age at BC diagnosis (HR per year increase: 1.02 (95%CI:1.01-1.02)) to be significantly associated with increased ovarian SPC risks (Table 5). We also found that females first diagnosed with BC after 2009 were at decreased risks of ovarian SPCs in comparison to those diagnosed with BC in 1995-99 (2010-14: HR: 0.77 (95%CI:0.67-0.88), 2015-2019: HR: 0.78 (95%CI:0.65-0.93)).

We also observed significant evidence that females from the IMD MDQ were at elevated ovarian SPC risks compared to females from the LDQ (HR: 1.21 (95%CI:1.07-1.38)) and that ovarian SPC risks were increased following a grade 3, rather than a grade 1, initial BC (HR: 1.39 (95%CI:1.21-1.60)).

#### Treating age at first breast cancer diagnosis as a 5-year categorical variable

We fitted two Cox models respectively assessing the influence of age at first breast cancer diagnosis on non-breast and contralateral breast SPC risks in females. In each model, age was fit as a categorical variable with the following categories: Under 40, 40-44, 45-49, 50-54, 55-59, 60-64, 65-69, 70-74, 75-79, 80 and over, with under 40 taken as the reference category.

We found that increasing age band was associated with an increase in non-breast SPC risks, and a consistent and clear relationship between increasing age band and decreasing contralateral breast cancer risks. The hazard ratios for non-breast SPCs relative to age <40, were 1.39 (95%CI:1.27-1.52) for age 40-45, 1.86 (95%CI:1.72-2.02) for age 45-49, 2.43 (95%CI:2.25-2.63) for age 50-54, 3.24 (95%CI:3.00-3.51) for age 55-59, 3.99 (95%CI:3.69-4.31) for age 60-64, 4.84 (95%CI:4.48-5.23) for age 65-69, 5.61 (95%CI:5.19-6.07) for age 70-74, 6.33 (95%CI:5.84-6.85) for age 75-79, and 6.53 (95%CI:6.03-7.08) for age 80 or over. The hazard ratios for contralateral breast cancer were 0.79 (95%CI:0.73-0.86) for age 40-45, 0.72 (95%CI:0.66-0.77) for age 45-49, 0.72 (95%CI:0.67-0.77) for age 50-54, 0.74 (95%CI:0.69-0.79) for age 55-59, 0.68 (95%CI:0.63-0.74) for age 60-64, 0.62 (95%CI:0.57-0.67) for age 65-69, 0.64 (95%CI:0.59-0.70) for age 70-74, 0.56 (95%CI:0.50-0.61) for age 75-79, and 0.47 (95%CI:0.42-0.52) for age 80 or over.

*Note: All references, abbreviations, and figures correspond to those used in the main manuscript, unless marked with the prefix ‘S’ – this indicates a supplementary reference/abbreviation/figure.*


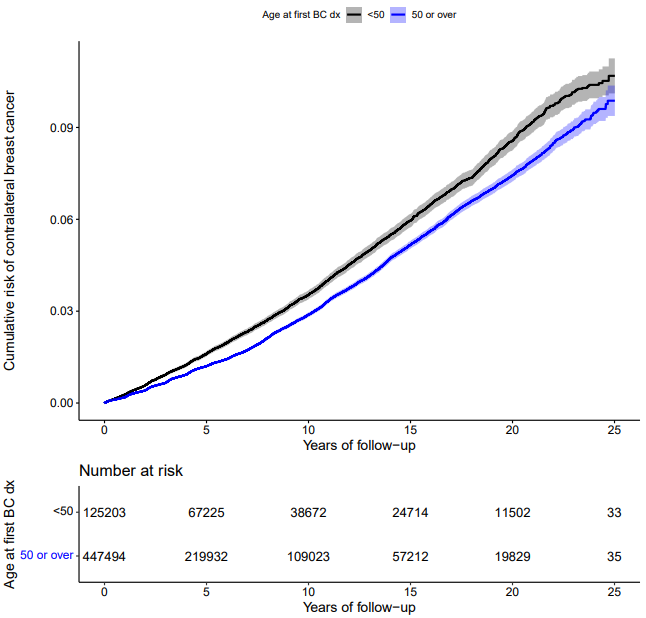

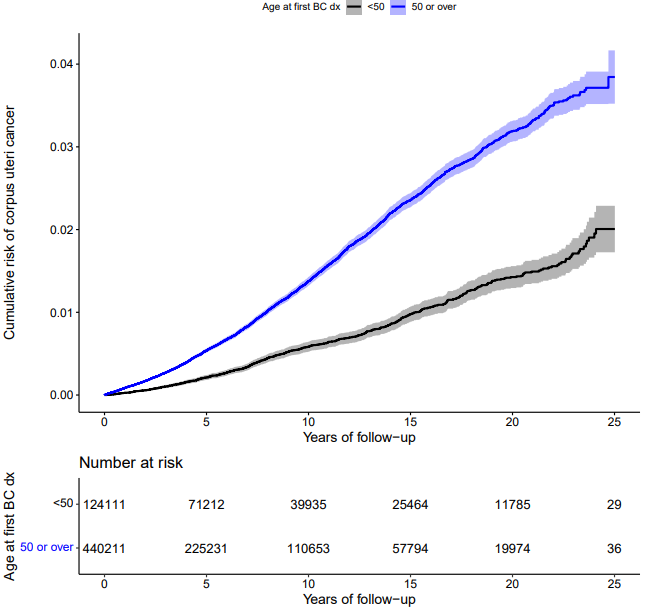

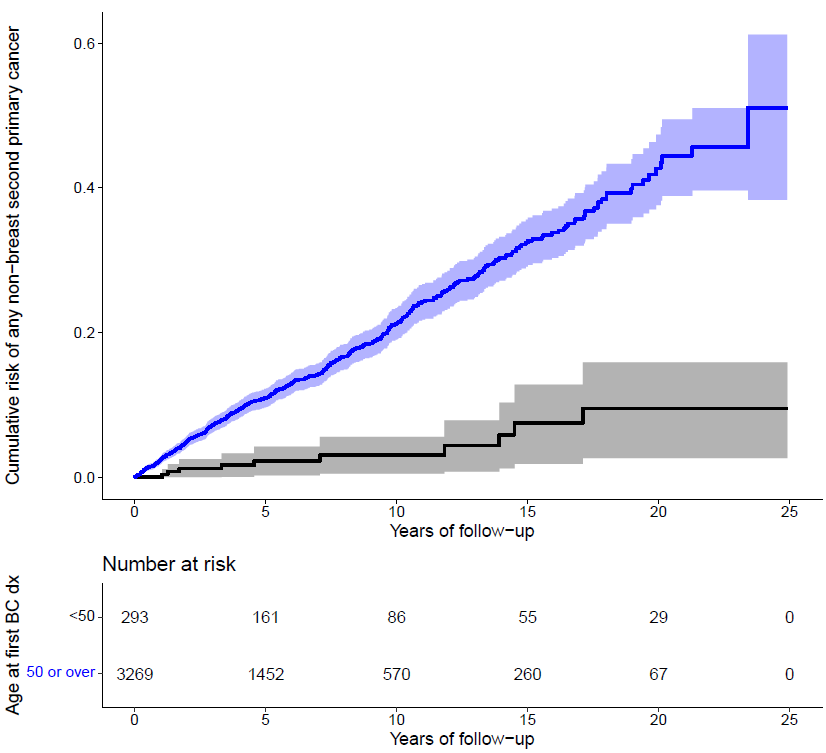

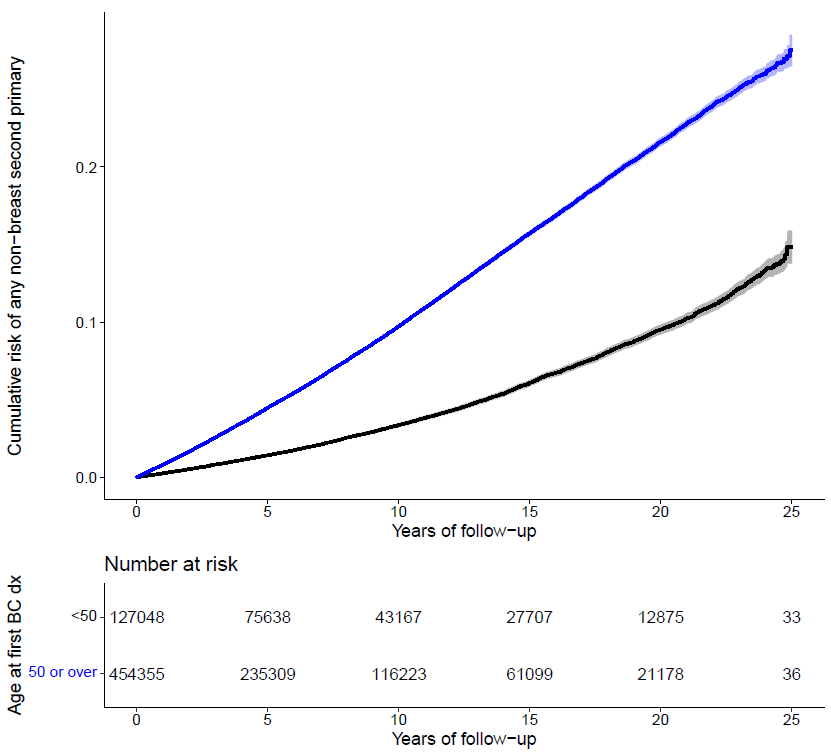
**Figure S1: Cumulative risks of second primaries, stratified by age at first breast cancer diagnosis**

5:

4:

3:

1:

2:

1: Cumulative risk of non-breast SPCs^1^ (female BC^2^ survivors)

2: Cumulative risk of CBC^3^ (female BC survivors)

3: Cumulative risk of endometrial SPCs (female BC survivors)

4: Cumulative risk of ovarian SPCs (female BC survivors)

5: Cumulative risk of non-breast SPCs (male BC survivors)

Black/grey scheme: Aged <50y^4^ at first BC diagnosis

Blue/light blue scheme: Aged >=50y at first BC diagnosis

1: Second Primary Cancer, 2: Breast Cancer, 3: Contralateral Breast Cancer, 4: Years

*Note: We do not present a cumulative risk curve for CBC in males, due to low numbers of events.*


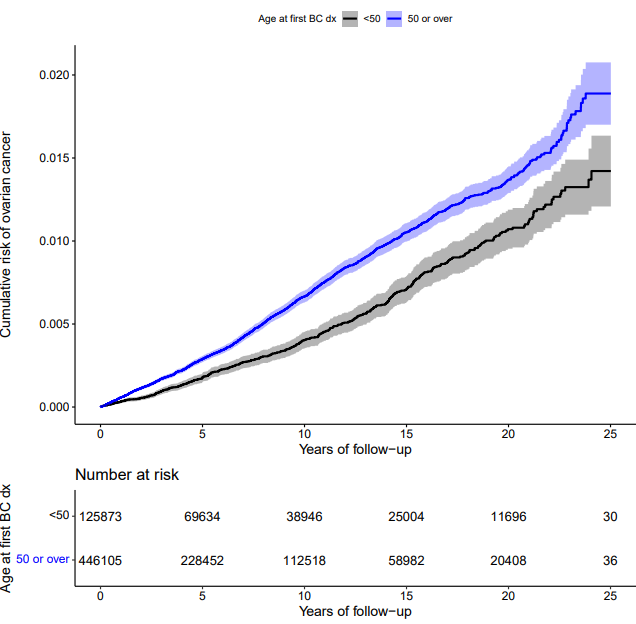


**Figure S2: Forest plots of standardized incidence ratios for second primaries in male and female breast cancer survivors**

**Female breast cancer survivors**

**Male breast cancer survivors**


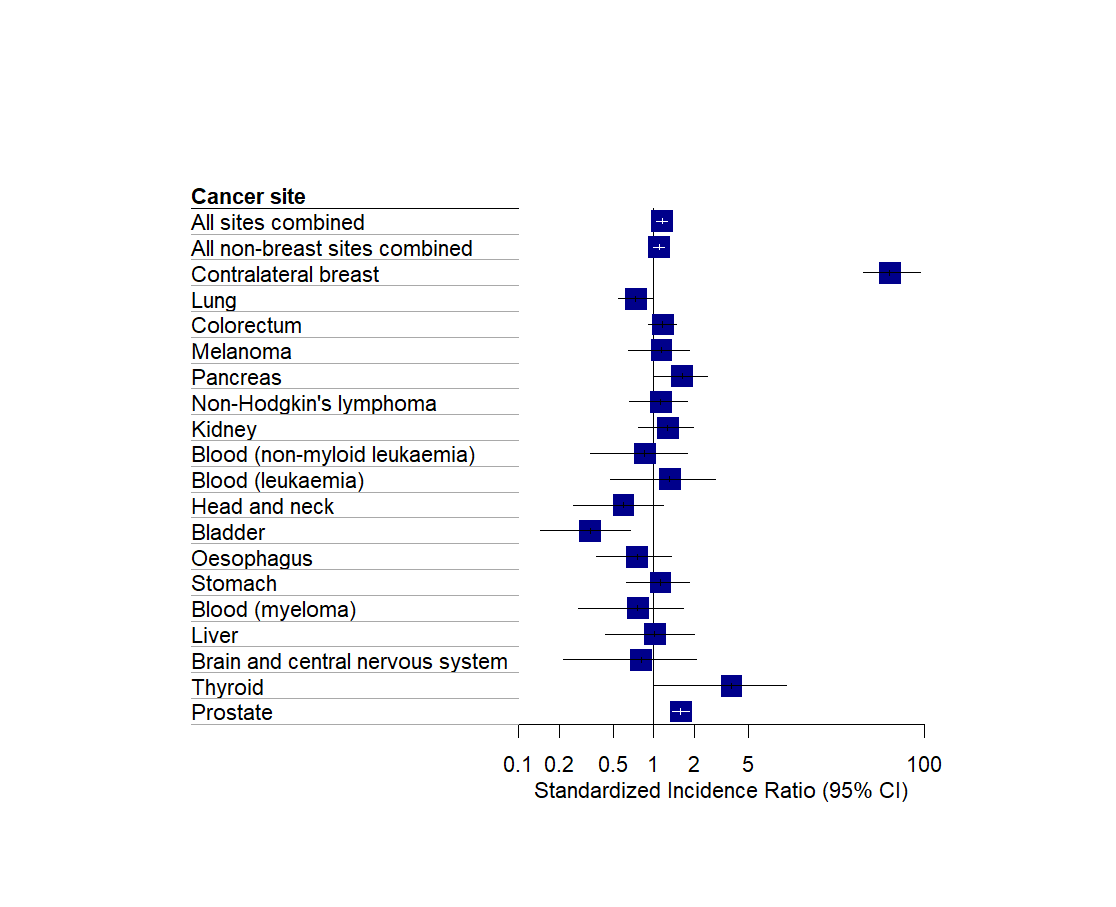

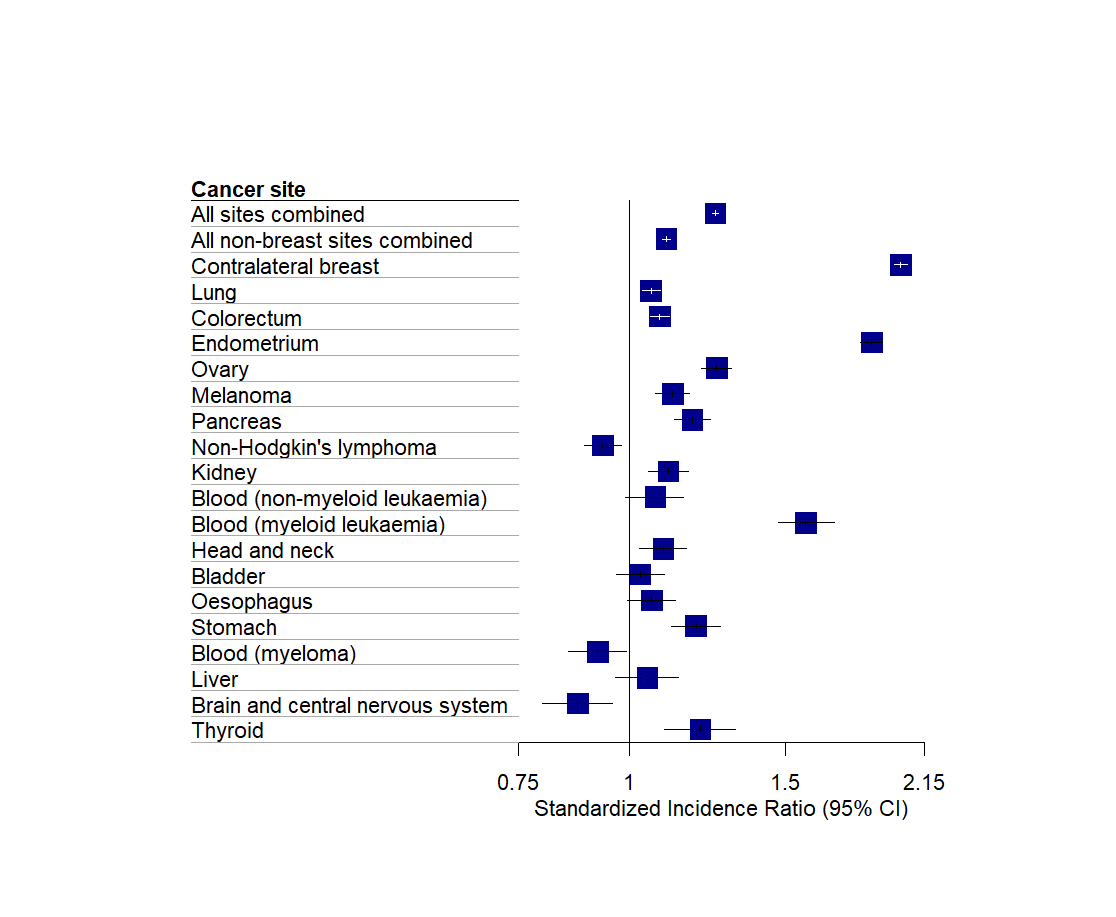


**Figure S3: Forest plots of standardized incidence ratios for second primaries by age at first breast cancer diagnosis in female breast cancer survivors**

**Age 50 or over**

**Under age 50**


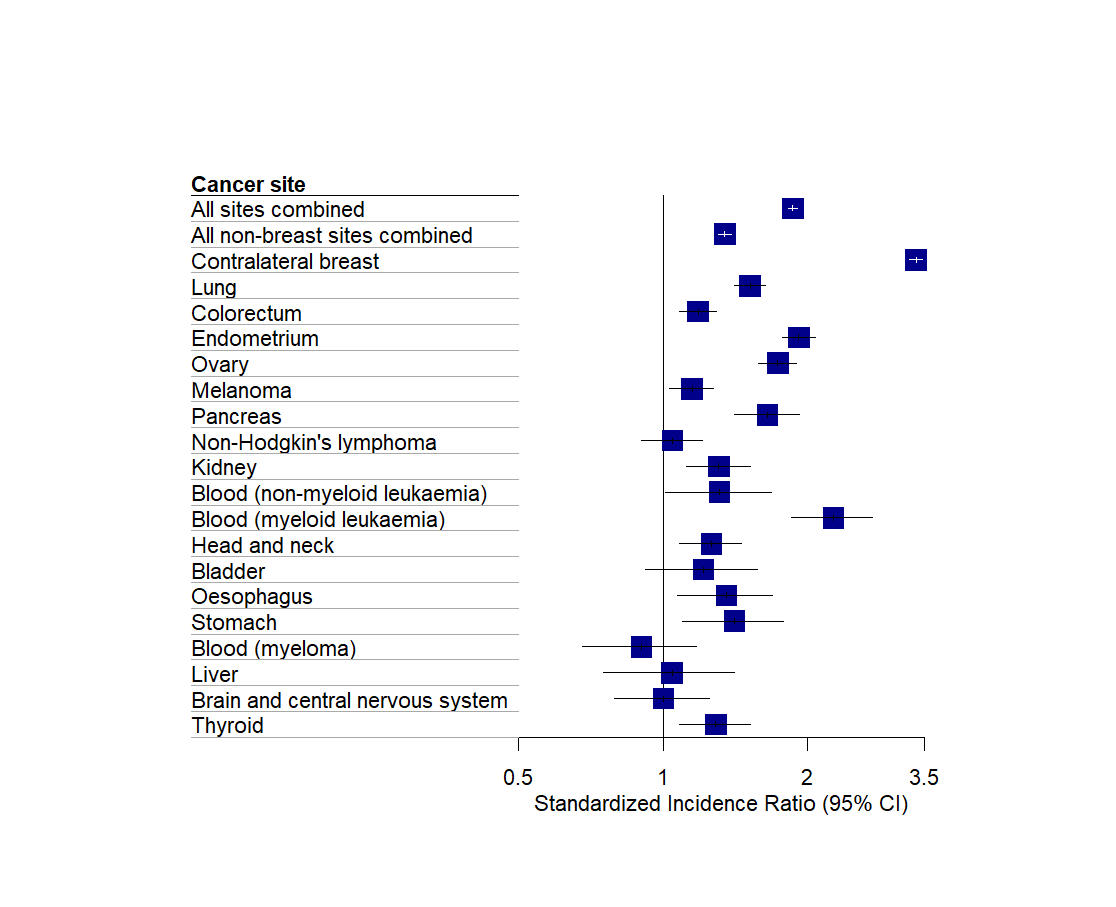

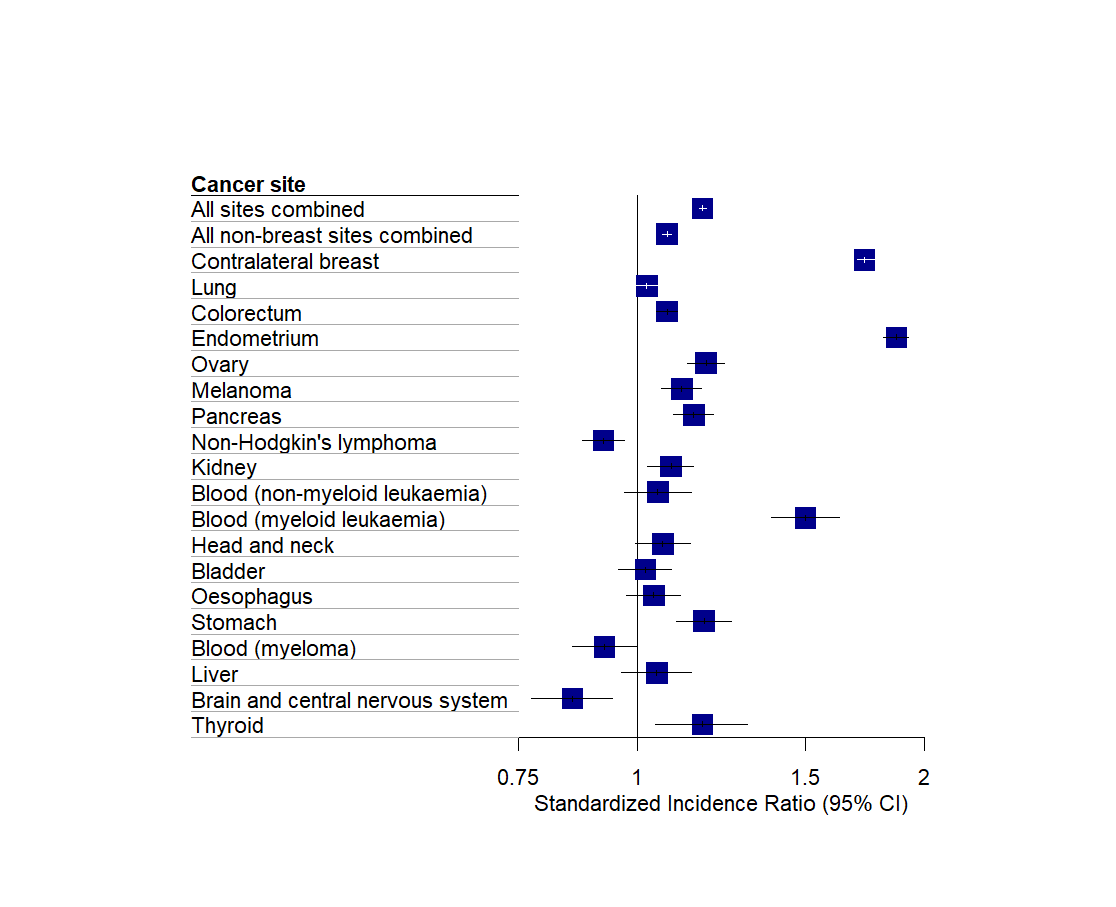


**Figure S4: Forest plots of standardized incidence ratios for second primaries by socioeconomic status of region lived in at time of first breast cancer diagnosis in females**


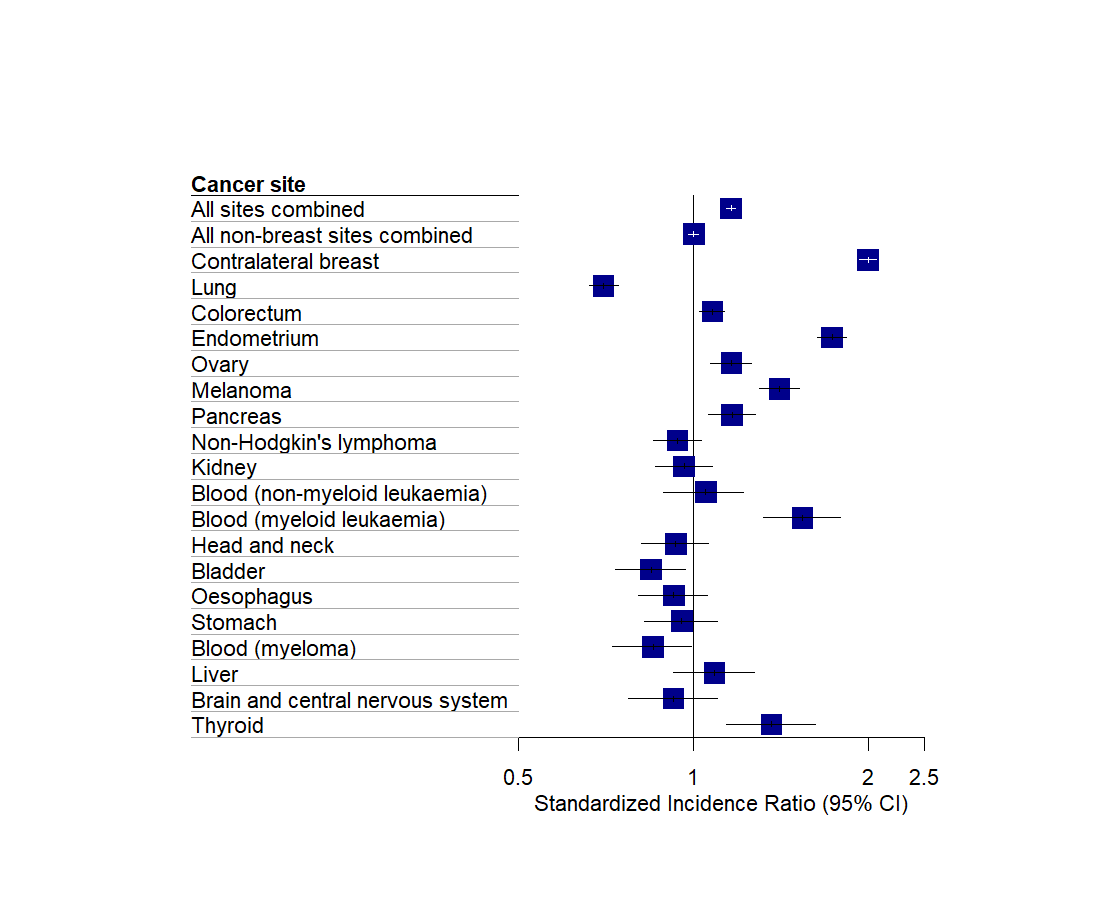


**Indices of Multiple Deprivation quintile 1 (most deprived)**

**Indices of Multiple Deprivation quintile 2 (least deprived)**


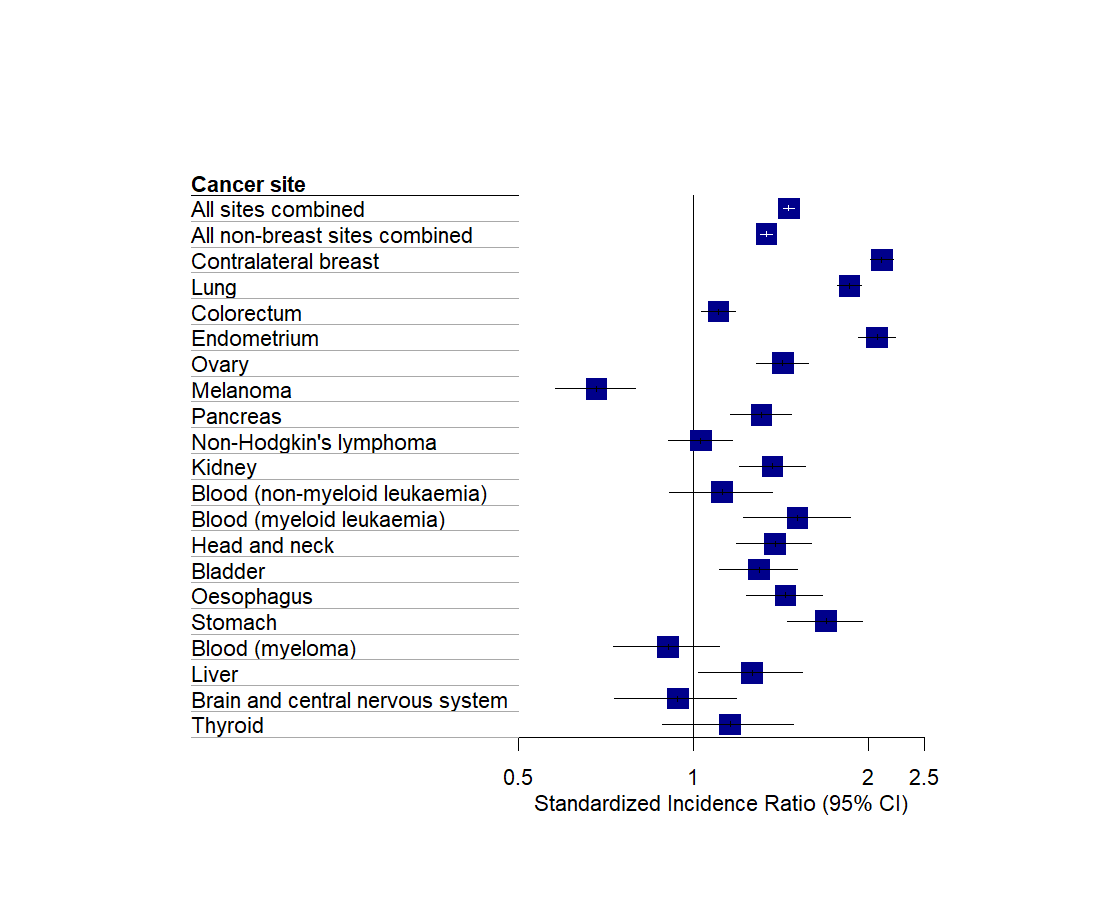


# Supplementary references

S1: White IR, Royston P. Imputing missing covariate values for the Cox model. *Stat Med.* 2009; **28(15):** 1982-98.

S2: Dowle M, Srinivasan A (2023). data.table: Extension of ‘data.frame’. R package version 1.14.8, <https://CRAN.R-project.org/package=data.table>

S3: R Special Interest Group on Databases (R-SIG-DB), Hadley Wickham and Kirill Müller (2021). DBI: R Database Interface. R package version 1.1.2, <https://CRAN.R-project.org/package=DBI>

S4: Hadley Wickham, Romain François, Lionel Henry and Kirill Müller (2021). dplyr: A Grammar of Data Manipulation. R package version 1.0.7, <https://CRAN.R-project.org/package=dplyr>

S5: Stevenson M, Sargeant E (2023). epiR: Tools for the Analysis of Epidemiological Data. R package version 2.0.62, <https://CRAN.R-project.org/package=epiR>

S6: Hadley Wickham (2021). forcats: Tools for Working with Categorical Variables (Factors). R package version 0.5.1, <https://CRAN.R-project.org/package=forcats>

S7: Schmidt D, Chen W (2017). “getPass: Masked User Input.” R package version 0.2-2, < https://CRAN.R-project.org/package=getPass>

S8: Stef van Buuren, Karin Groothuis-Oudshoorn (2011). mice: Multiple Imputation by Chained Equations in R. Journal of Statistical Software, 45(3), 1-67. DOI 10.18637/jss.v045.io3.

S9: Garrett Grolemund, Hadley Wickham (2011). Dates and Times Made Easy with lubridate. Journal of Statistical Software, 40(3), 1-25. URL https://www.jstatsoft.org/v40/i03/.

S10: Hadley Wickham and Jennifer Bryan (2022). readxl: Read Excel Files. R package version 1.4.0, <https://CRAN.R-project.org/package=readxl>

S11: Urbanek S (2021). rJava: Low-Level R to Java Interface. R package version 1.0-6, <https://CRAN.R-project.org/package=rJava>

S12: Urbanek S (2022). RJDBC: Provides Access to Databases Through the JDBC Interface. R package version 0.2-10, <https://CRAN.R-project.org/package=RJDBC>

S13: Therneau T (2021). A Package for Survival Analysis in R. R package version 3.2-13, <https://CRAN.R-project.org/package=survival>.

S14: Alboukadel Kassambara, Marcin Kosinski and Przemyslaw Biecek (2021). survminer: Drawing Survival Curves using 'ggplot2'. R package version 0.4.9, <https://CRAN.R-project.org/package=survminer>

S15: Grosjean, Ph. (2023). SciViews::R. UMONS, Mons, Belgium. URL: <https://sciviews.r-universe.dev/>.

S16: R Core Team (2021). R: A language and environment for statistical computing. R Foundation for Statistical Computing, Vienna, Austria. URL <https://www.R-project.org/>.
